# Genomic surveillance meets clinical practice: rule-based individualized treatment prediction for gonorrhoea using SensiTyper

**DOI:** 10.1038/s41467-026-79037-z

**Published:** 2026-10-30

**Authors:** Daniel Golparian, Leonor Sánchez-Busó, Magnus Unemo

**Affiliations:** 1 WHO Collaborating Centre for Gonorrhoea and other Sexually Transmitted Infections, Department of Laboratory Medicine, Faculty of Medicine and Health, Örebro University, Örebro, Sweden; 2 Joint Research Unit ‘Infection and Public Health’, FISABIO-University of Valencia, Institute for Integrative Systems Biology (I2SysBio), Valencia, Spain; 3 CIBERESP, ISCIII, Madrid, Spain; 4 Institute for Global Health, University College London (UCL), London, UK

**Keywords:** Molecular medicine, Antimicrobial resistance

## Abstract

Extensively drug-resistant (XDR) *Neisseria gonorrhoeae* strains threaten the current empirical gonorrhoea treatment. We designed and analytically validated SensiTyper, a rule-based framework that predicts antimicrobial susceptibility from whole-genome sequencing data and generates susceptibility-guided individualized treatment recommendations. SensiTyper was applied to 13,701 gonococcal genomes from 68 countries in 1928-2024, including cultured isolates and clinical molecular samples, and benchmarked predictions against previous genomic and phenotypic data. Susceptibility predictions for ceftriaxone, ciprofloxacin, spectinomycin, and zoliflodacin had 98.9-100% sensitivity and specificity. Azithromycin susceptibility predictions had 92.5% sensitivity and 97.1% specificity, and gepotidacin susceptibility prediction 100% sensitivity, but 0% specificity, illustrating that low-level phenotypic gepotidacin resistance is not caused by the only known resistance determinants (GyrA A92T plus ParC D86N). The SensiTreat module integrates antimicrobial susceptibility profiles into sequential treatment logic, providing susceptibility-guided individualized treatment recommendations while also enabling alternative stewardship strategies prioritizing, e.g., ciprofloxacin. SensiTyper flags XDR isolates for expert follow-up, test-of-cure and comprehensive contact tracing. In conclusion, SensiTyper is a robust and adaptable approach for gonococcal antimicrobial susceptibility prediction, individualized gonorrhoea treatment and antimicrobial stewardship.

## Abstract (web summary)

Golparian et al. designed and validated (13,701 global gonococcal genomes) SensiTyper, a rule-based framework predicting antimicrobial susceptibility from whole-genome sequencing data and susceptibility-guided individualized gonorrhoea treatment.

## Introduction

N*eisseria gonorrhoeae*, causing gonorrhoea, has evolved, acquired, and adapted antimicrobial resistance (AMR) mechanisms to every antimicrobial introduced for treatment since the mid-1930s, and more recently to the last remaining treatment option, the extended-spectrum cephalosporin (ESC) ceftriaxone^1–4^. This has resulted in a stable population of multidrug-resistant (MDR) strains in many countries^2–10^, sporadic ceftriaxone treatment failures worldwide^11–14^, and in the most recent decade, extensively drug-resistant (XDR) strains that are resistant to both ceftriaxone and azithromycin, the backbone of the current first-line therapy^3,9,12,15–20^.

International gonorrhoea treatment guidelines currently recommend empiric first-line therapy consisting of a high-dose ceftriaxone monotherapy (0.5–1 g intramuscularly (IM) or intravenously) or ceftriaxone 0.5–1 g IM plus azithromycin 1–2 g orally^2,21^. Alternative treatment regimens include spectinomycin (2 g IM) plus azithromycin (2 g orally), ciprofloxacin (500 mg orally) for confirmed ciprofloxacin-susceptible infections (based on molecular *gyrA* test or phenotypic antimicrobial susceptibility testing (AST)), or gentamicin (240 mg IM) plus azithromycin (2 g orally) in cases of hypersensitivity to beta-lactam antimicrobials, ceftriaxone resistance, or ceftriaxone treatment failures^2,21^. New antimicrobials like zoliflodacin^22,23^, targeting GyrB, and gepotidacin^24^, targeting GyrA and ParC, may soon be implemented in the gonorrhoea treatment. These antimicrobials received US FDA approval for the treatment of uncomplicated urogenital gonorrhoea in December 2025^25^.

Antimicrobial resistance in *N. gonorrhoeae* has evolved through a variety of AMR mechanisms^2,26^. For ceftriaxone, resistance is driven by mosaic *penA* alleles that encode mosaic penicillin-binding protein 2 (PBP2) with A311V and V316 amino acid alterations^9,27,28^. Azithromycin resistance results from 23S rRNA mutations (notably in A2059 and C2611)^29,30^ or enhanced efflux mediated especially by mosaic or semi-mosaic MtrD^31–33^, which may be partially reversed by disrupted *mtrC*^34^. Ciprofloxacin resistance is associated with mutations in *gyrA* and *parC*^35^, especially the GyrA S91F mutation. Resistance to spectinomycin is caused by 16S rRNA^36^ or *rpsE* mutations^37^. For zoliflodacin, increased minimum inhibitory concentrations (MICs) are associated with amino acid alterations in GyrB D429 or K450^38–40^. Resistance to gepotidacin is caused by concomitant GyrA A92T and ParC D86N substitutions^23,41–44^.

Whole genome sequencing (WGS), including AMR prediction based on AMR-conferring mutations, has emerged as an alternative to culture-based methods for AST in *N. gonorrhoeae*. Eyre et al.^45^ applied multivariate linear regression to WGS data, achieving MIC predictions within ±1 doubling dilution for 93% of isolates across five key antimicrobials. Demczuk et al.^46^ derived predictive equations for MICs based on curated genetic AMR determinants and a multivariate linear regression model across multiple national collections. Their approaches showed high reproducibility and specificity, particularly for ciprofloxacin and ESCs; however, inherent limitations of rule-based models when applied to complex phenotypes like azithromycin resistance, where multiple overlapping mechanisms may contribute additively or epistatically for MIC predictions, were highlighted. Direct prediction of antimicrobial susceptibility or AMR categories, instead of MICs, may offer more practical value and robustness for clinical implementation. Importantly, these previous prediction studies^45,46^ were conducted prior to the emergence of globally circulating ceftriaxone-resistant strains carrying PBP2 A311V and V316 alterations, such as mosaic *penA*−60^8,9,12,13,15–20,28,47–49^ and *penA*−237^9,20,49,50^, and the widespread MtrD mosaic/semi-mosaic variants causing low-level azithromycin resistance^31–33^. Consequently, these key AMR determinants are absent from their models, limiting their applicability to current clinical AMR scenarios. It is imperative to continuously update the WGS-based AST that must meet rigorous accuracy standards, particularly regarding very major errors (VMEs), which represent the highest risk for inappropriate clinical decisions, i.e., predicting antimicrobial susceptibility for an AMR isolate. Hicks et al.^51^ conducted a meta-analysis of multiple large datasets from *N. gonorrhoeae*, *Klebsiella pneumoniae*, and *Acinetobacter baumannii*, applying machine learning models, including random forest and set covering machines, to predict AMR phenotypes from WGS data. Ciprofloxacin resistance could be predicted with high accuracy; however, model performance was substantially lower for azithromycin. The model performance was also sensitive to sampling bias, with training datasets that failed to capture population-specific AMR determinants leading to poor generalization across temporally and geographically diverse datasets. However, antimicrobial susceptibility is frequently easier to predict than AMR, particularly when AMR involves multiple or unknown genetic determinants. This has important clinical implications, as antimicrobial susceptibility may be quickly inferred more reliably from the absence of key AMR mutations and promptly guide individualized therapy. Culture-based diagnostics, including AST, have been increasingly replaced by nucleic acid amplification test (NAAT) diagnostics that rarely provide AMR information. WGS has emerged as a comprehensive alternative, capable of detecting known AMR determinants across the genome sequenced directly from clinical NAAT samples^52^, which remains in rapid further development. Hopefully, real-time WGS for gonococcal AMR prediction directly from clinical NAAT samples will soon be implemented in clinical routine diagnostics to inform treatment. However, translating genomic data into actionable treatment recommendations should be benchmarked against clinical AST breakpoints or epidemiological cut-off values (ECOFFs), and quality control (QC) standards, harmonized interpretive frameworks, and curated databases of AMR determinants are imperative^53^.

Herein, we describe the development and analytical validation of SensiTyper, a curated rule-based tool that interprets WGS data to predict antimicrobial susceptibility in *N. gonorrhoeae* and recommend susceptibility-guided individualized gonorrhoea treatment. Unlike previous models that predicted antimicrobial MICs through statistical inference or machine learning, SensiTyper applies transparent, biologically interpretable rules to directly infer categories of antimicrobial susceptibility or AMR based on curated genetic AMR determinants. SensiTyper was analytically validated, examining 13,701 *N. gonorrhoeae* genomes (13,633 isolates with validated antimicrobial MICs) from diverse temporal and geographical settings, and SensiTyper’s susceptibility predictive performance was benchmarked against established clinical AMR breakpoints or ECOFF (for azithromycin).

## Results

### *Neisseria gonorrhoeae* genomic dataset and antimicrobial susceptibility profiles

We analysed 13,701 *N. gonorrhoeae* genomes (isolates were available for AST of 13,633 isolates), including 13,133 global genomes from 32 previously published studies spanning 68 countries and almost a century of sampling (1928–2024) (Supplementary Data, Supplementary Fig 1) and 568 genomes from contemporary isolates obtained in Sweden (2017-2024) and sequenced in the present study, that is, to validate our clinical routine diagnostic workflow including gonococcal culture, promptly followed by WGS and analysis with SensiTyper.

**Fig. 1:**
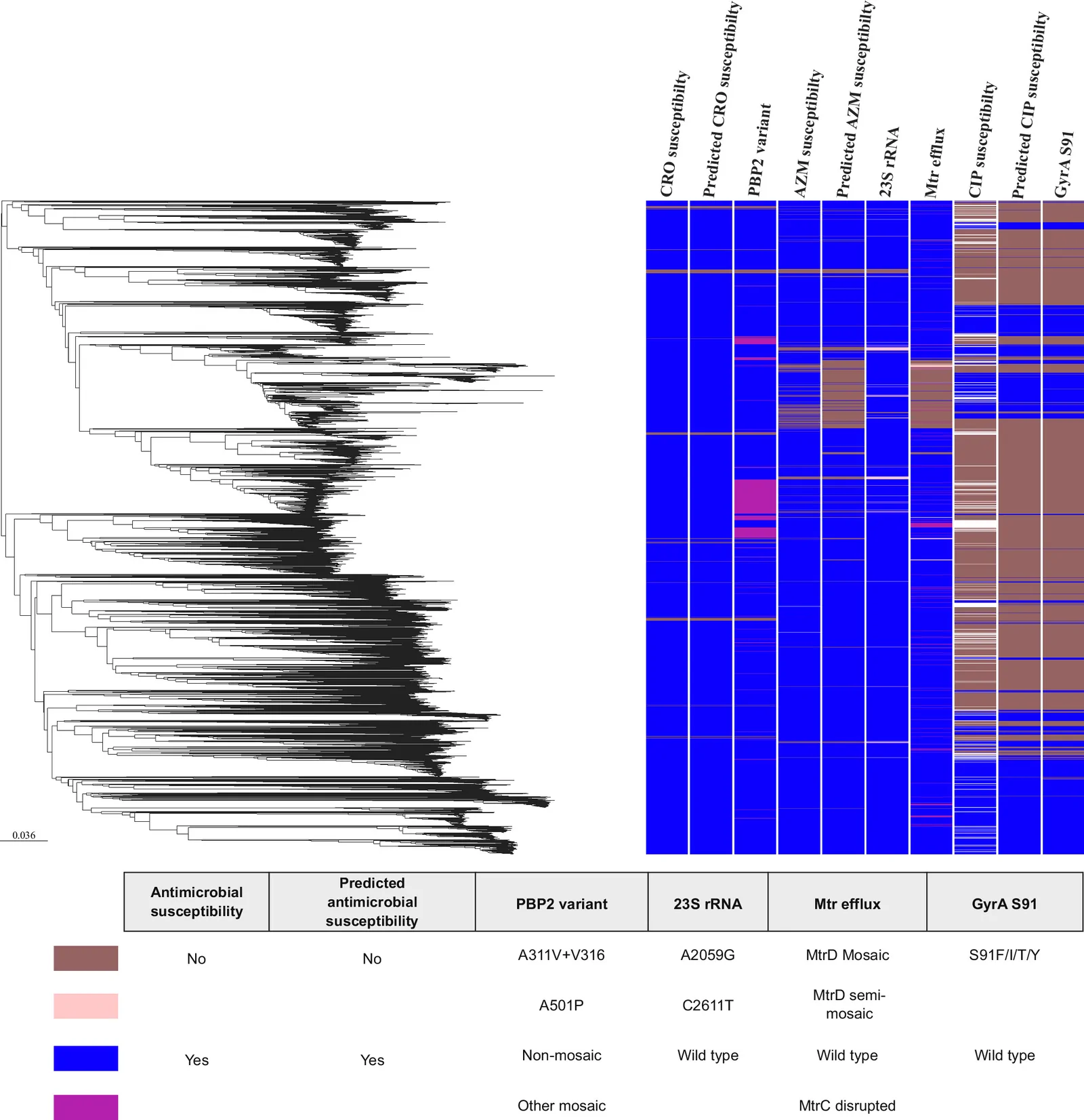
A neighbour-joining tree generated in Ridom Seqsphere+(v10.0.1)^67^ using cgMLST allele profiles for 13,633 *N. gonorrhoeae* isolates (Supplementary Data, Supplementary Fig. 1) and 50 *N. gonorrhoeae-*positive NAAT samples (with paired gonococcal isolates (*n* = 58)). Annotation tracks adjacent to the tree summarize phenotypic antimicrobial susceptibility based on MIC testing, genomic predictions of antimicrobial susceptibility generated using the SensiTyper, and key antimicrobial resistance determinants. White colour denotes missing data. Source data are provided as a Source Data file. CRO ceftriaxone, PBP2 penicillin-binding protein 2, AZM azithromycin, CIP ciprofloxacin.

The majority of isolates were susceptible to ceftriaxone (97.4%), azithromycin (92.2%), and spectinomycin (99.8%), while only 35.0% of isolates were susceptible to ciprofloxacin (Table 1, Fig. 1). For zoliflodacin and gepotidacin, 99.8% and 95.0% of isolates, respectively, were susceptible. Eighty-six isolates were defined as XDR isolates (resistant to ceftriaxone, cefixime, azithromycin, and ciprofloxacin).

**Table 1:** Antimicrobial susceptibility and resistance of all examined *Neisseria gonorrhoeae* isolates (Supplementary Data, Supplementary Fig. 1)

Antimicrobial	Susceptible, % (no. susceptible/examined)	Resistant, % (no. resistant/examined)	MIC range (mg/L)
Ceftriaxone^a^	97.4 (13,282/13,633)	2.6 (351/13,633)	0.001–2
Azithromycin^a^	92.2 (12,570/13,633)	7.8 (1063/13,633)	0.008–>256
Ciprofloxacin^a^	35.0 (4109/11,741)	64.7 (7591/11,741)	0.001–>32
Spectinomycin^a^	99.8 (1363/1366)	0.2 (3/1366)	2–>1024
Zoliflodacin^b^	99.8 (3226/3231)	N/A^c^	0.001–8
Gepotidacin^d^	95.0 (2789/2937)	0.2 (6/2937)	0.016–4

^a^Ceftriaxone: susceptible (S), MIC ≤ 0.125 mg/L and resistant (R), MIC > 0.125 mg/L; azithromycin: S, MIC ≤ 1 mg/L and R, MIC > 1 mg/L; ciprofloxacin: S, MIC ≤ 0.032 mg/L, susceptible, increased exposure (I), MIC = 0.064 mg/L, and R, MIC > 0.064 mg/L; and spectinomycin: S, MIC ≤ 64 mg/L and R, MIC > 64 mg/L (EUCAST v16.0; https://www.eucast.org/bacteria/clinical-breakpoints-and-interpretation/clinical-breakpoint-tables/).

^b^S, MIC ≤ 0.25 mg/L (https://www.fda.gov/drugs/development-resources/antibacterial-susceptibility-test-interpretive-criteria).

^c^N/A, not applicable. No resistance breakpoint has been stated for zoliflodacin (https://www.fda.gov/drugs/development-resources/antibacterial-susceptibility-test-interpretive-criteria).

^d^S, MIC ≤ 1 mg/L, I, MIC = 2 mg/L, and R, MIC ≥ 4 mg/L (https://www.fda.gov/drugs/development-resources/antibacterial-susceptibility-test-interpretive-criteria).

### Concordance of SensiTyper’s key genetic loci detection with previous studies

In total, 12,787 (97.4%) of the previously published 13,133 genomes (Supplementary Data, Supplementary Fig. 1) had been earlier analysed for all key genetic AMR determinants, with the exception of MtrD mosaicism/semi-mosaicism, which had been examined in 9099 (69.3%) isolates. All genomes were re-analysed with SensiType, using ARIBA^54^ as the central module for detection of AMR determinants, prior to treatment prediction using SensiTreat. For ceftriaxone, the wild-type loci (PBP2 A311, V316, and A501) were correctly identified by SensiType among the 12,787 isolates, except for two genomes in which SensiType detected the PBP2 A311V alteration, whereas previous studies had missed this mutation and designated them as *penA* wild type. Neither genome carried a PBP2 V316 mutation, and both remained susceptible to ceftriaxone. These discrepancies were resolved, and SensiType confirmed the correct calls. For azithromycin, resistance determinants at 23S rRNA positions A2059 and C2611, and MtrD mosaic/semi-mosaic variants were assessed. One azithromycin-resistant isolate carrying 23S rRNA A2059G was reported as 23S rRNA wild type in prior studies, but it was correctly identified as having 23S rRNA A2059G in all four alleles by SensiType. However, one genome with 23S rRNA C2611T in one of the four alleles was incorrectly classified as 23S rRNA wild type by SensiType. SensiType calls of the key determinant for ciprofloxacin susceptibility, GyrA S91, were consistent with previous data, except for ten genomes that had been designated GyrA wild type in earlier studies but were correctly identified by SensiType as carrying the S91Y/T mutations. No discrepancies were found for spectinomycin, zoliflodacin and gepotidacin.

All discrepancies were manually extracted, quality-controlled, and the reads were visually inspected to resolve the discrepancies.

### Prediction of antimicrobial susceptibility with SensiType

**Fig. 2:**
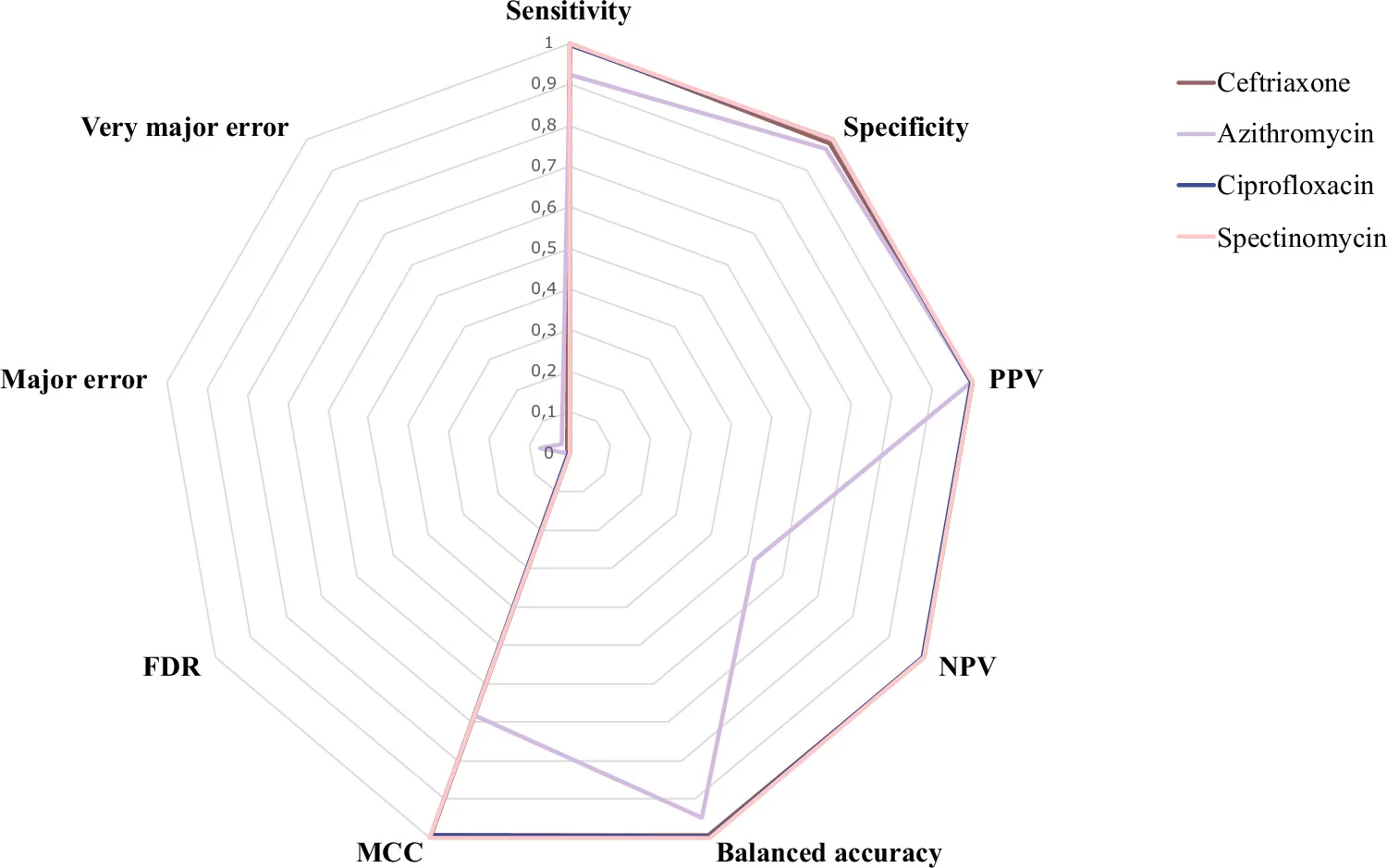
Radar plot of performance metrics for the prediction of susceptibility to the four antimicrobials with clinical breakpoints (EUCAST v16.0; https://www.eucast.org/bacteria/clinical-breakpoints-and-interpretation/clinical-breakpoint-tables/) that are included in the current gonorrhoea treatment guidelines^2,21^. Comparison of genomic predictions of antimicrobial susceptibility from SensiType with MIC-based antimicrobial susceptibility testing. Radar plots show sensitivity, specificity, positive predictive value (PPV), negative predictive value (NPV), balanced accuracy, Matthews correlation coefficient (MCC), false discovery rate (FDR), major error, and very major error in the prediction of susceptibility to ceftriaxone, azithromycin, ciprofloxacin, and spectinomycin. The lower sensitivity, NPV and MCC, as well as a higher level of major errors in the prediction of susceptibility to azithromycin, are clearly shown. Source data in Table 2.

To initially benchmark the ability of the SensiType module to predict phenotypic antimicrobial susceptibility, we applied it to all genomes for which validated MIC data and clinical breakpoints (EUCAST v16.0; https://www.eucast.org/bacteria/clinical-breakpoints-and-interpretation/clinical-breakpoint-tables/) were available. Genomic predictions were compared with MIC-based results for these four antimicrobials that are included in current gonorrhoea treatment guidelines^2,21^, i.e., ceftriaxone (*n* = 13,633), azithromycin (*n* = 13,633), ciprofloxacin (*n* = 11,741), and spectinomycin (*n* = 1366) (Fig. 2).

For ceftriaxone, predictions were all 98.9–100%; sensitivity (100.0%, CI 95%: 99.9–100%), specificity (98.9%, CI 95%: 97.1–99.6%), positive predictive value (PPV) (100.0%, CI 95%: 99.9–100%), and negative predictive value (NPV) (100.0%, CI 95%: 98.9–100%). However, the majority of isolates were susceptible to ceftriaxone, creating an imbalanced dataset. To account for this, we calculated the Matthews correlation coefficient (MCC), balance accuracy (BA), and false discovery rate (FDR), which were 0.994, 99.4%, and 0.03%, respectively (Table 2).

**Table 2:** Performance of SensiType predictions of antimicrobial susceptibility compared with phenotypic MIC-based antimicrobial susceptibility testing for six antimicrobials

	Ceftriaxone (CI 95%)	Azithromycin (CI 95%)	Ciprofloxacin (CI 95%)	Spectinomycin (CI 95%)	Zoliflodacin (CI 95%)	Gepotidacin (CI 95%)
Total analysed	13,633	13,633	11,741	1366	3231	2937
True Positive	13,282	11,625	4131^a^	1363	3226	2931^b^
True Negative	347	1032	7578	3	5	0
False Positive	4	31	13	0	0	6
False Negative	0	945	19^a^	0	0	0
Sensitivity (%)	100.0 (99.9–100)	92.48 (92.00–92.93)	99.54 (99.29–99.71)	100.0 (99.71–100)	100.0 (99.88–100)	100.0 (99.87–100)
Specificity (%)	98.86 (97.11–99.56)	97.08 (95.89–97.94)	99.83 (99.71–99.90)	100.0 (43.85–100)^c^	100.0 (56.55–100)^c^	0.0 (0–39.03)
Positive predictive value (PPV) (%)	99.97 (99.92–99.99)	99.73 (99.62–99.81)	99.69 (99.46–99.82)	100.0 (99.72–100)	100.0 (99.88–100)	99.80 (99.56–99.91)
Negative predictive value (NPV) (%)	100.0 (98.91–100)	52.20 (50.00–54.40)	99.75 (99.61–99.84)	100.0 (43.85–100)^c^	100.0 (56.55–100)^c^	N/A^d^
Balance accuracy (BA) (%)	99.43	94.78	99.69	100.0	100.0	50.0
Matthews correlation coefficient (MCC)	0.9941	0.6820	0.9940	1.0	1.0	0.0
False discovery rate (FDR) (%)	0.03 (0.01–0.08)	0.26 (0.18–0.38)	0.31 (0.18–0.54)	0.0 (0.00–0.28)	0.0 (0.00–0.12)	0.20 (0.09–0.45)
Major error rate (ME) (%)	0.0 (0.00–0.03)	7.52 (7.1–8.0)	0.46 (0.29–0.71)	0.0 (0.00–0.28)	0.0 (0.00–0.12)	0.0 (0.0–0.13)
Very major error rate (VME) (%)	1.14 (0.44–2.90)	2.92 (2.06–4.11)	0.17 (0.10–0.29)	0.0 (0.00–56.2)^c^	0.0 (0.00–43.4)^c^	100.0 (60.97–100)

Metrics include true and false classifications, predictive values, balanced accuracy, correlation coefficients, and error rates for ceftriaxone, azithromycin, ciprofloxacin, spectinomycin, zoliflodacin, and gepotidacin.

^a^Included 41 isolates with EUCAST’s ‘susceptible, increased exposure’ (reduced susceptibility) phenotype, which were considered as susceptible (possible to cure) in the performance calculations.

^b^Included 142 isolates with intermediate susceptibility phenotype (https://www.fda.gov/drugs/development-resources/antibacterial-susceptibility-test-interpretive-criteria), which were considered as susceptible (possible to cure) in the performance calculations.

^c^Nearly all isolates were susceptible, resulting in wide 95% confidence intervals.

^d^N/A, not applicable.

The inclusion of BA, MCC, and FDR ensured that predictive performance was assessed across both antimicrobial susceptible and non-susceptible groups, accounting for dataset imbalance while also capturing how often susceptibility predictions were incorrect. The ME and VME rates for ceftriaxone susceptibility prediction were 0.0% (CI 95%: 0.00–0.03%) and 1.1% (CI 95%: 0.4–2.9%), respectively. In contrast, azithromycin susceptibility prediction was more challenging. Genomic predictions showed high sensitivity of 92.5% (CI 95%: 92.0–92.9%) and specificity of 97.1% (CI 95%: 95.9–97.9%), with a PPV of 99.7% (CI 95%: 99.6–99.8%), but a markedly lower NPV of 52.2% (CI 95%: 50.0–54.4%), indicating that azithromycin-susceptible isolates were misclassified as azithromycin resistant. The MCC was 0.68, BA 94.8% and FDR 0.26%, indicating reduced concordance compared to ceftriaxone. The ME rate was 7.5% (CI 95%: 7.1–8.0%), and the VME rate was 2.9% (CI 95%: 2.1–4.1%) for azithromycin susceptibility predictions, with 31 azithromycin-resistant isolates predicted to be azithromycin susceptible. The azithromycin MICs of these isolates were reported as 2 mg/L (*n* = 27), 4 mg/L (*n* = 3), and 8 mg/L (*n* = 1) with wild-type MtrD and 23S rRNA. Unfortunately, these isolates were not available for retesting of the azithromycin MICs in the present study. For ciprofloxacin, susceptibility predictions were highly concordant with phenotypic results. Sensitivity was 99.5% (CI 95%: 99.3–99.7%) and specificity 99.8% (CI 95%: 99.7–99.8%), with a PPV of 99.7% (CI 95%: 99.5–99.8%) and an NPV of 99.8% (CI 95%: 99.6–99.8%). The MCC was 0.994, BA 99.7% and FDR 0.31%. The ME rate was 0.5% (CI 95%: 0.3–0.7%), corresponding to 19 false negatives, and the VME rate was 0.2% (CI 95%: 0.1–0.3%), with 13 false positive predictions. However, 16 of the false negatives had EUCAST’s ‘susceptible, increased exposure’ (reduced susceptibility) phenotype (MIC = 0.064 mg/L) and three had MIC = 0.032 mg/L. The VME isolates (*n* = 13) had MIC = 0.125 mg/L (*n* = 12) and MIC = 0.25 mg/L (*n* = 1), and no mutations in GyrA S91F, while four isolates had mutations in GyrA D95. For spectinomycin, all performance metrics, including sensitivity, specificity, PPV, NPV, MCC, and BA, were 100%, with no errors observed among the 1363 (99.8%) spectinomycin-susceptible isolates. For the new antimicrobial zoliflodacin^22,23^, hopefully soon included in international gonorrhoea treatment guidelines, the susceptibility predictions showed complete concordance with phenotypic zoliflodacin testing among 3226 (99.8%) susceptible isolates. However, nearly all isolates were susceptible to spectinomycin and zoliflodacin, and the exceedingly low number of resistant isolates results in wide confidence intervals for specificity, NPV, and VME rates. For the other new antimicrobial soon included in international gonorrhoea treatment guidelines, i.e., gepotidacin^24^, the sensitivity and PPV were 100% and 99.8%, respectively, and no ME were identified in the susceptibility prediction among 2,937 isolates; however, the specificity was 0% and the VME rate was 100% (Table 2). Accordingly, six isolates were resistant to gepotidacin (MIC = 4 mg/L) using the resistance breakpoint recently stated by the US FDA (MIC ≥ 4 mg/L; https://www.fda.gov/drugs/development-resources/antibacterial-susceptibility-test-interpretive-criteria), however, the genomic prediction identified all these isolates as susceptible to gepotidacin. The reason for this is that gonococcal isolates with low-level phenotypic resistance to gepotidacin using the new resistance breakpoint do not contain the only known determinants for gepotidacin resistance in *N. gonorrhoeae* (GyrA A92T plus ParC D86N substitutions)^24,42–44^.

### Susceptibility-guided individualized treatment recommendations using SensiTreat

In the SensiTreat module, treatment assignment is performed in a sequential mode, where antimicrobial regimens are recommended only for isolates predicted to be susceptible, and ineffective options are excluded stepwise until a treatable option is identified. As a result, metrics that rely on explicit “true negatives”, such as specificity, are less informative in this context and are not reported. Instead, performance is evaluated using sensitivity, predictive values, and error rates, together with coverage fraction to capture the proportion of isolates that receive the predicted treatment at each sequential step (Table 3).

**Table 3:** Performance of SensiTreat susceptibility-guided individualized treatment recommendation strategies compared with phenotypic MIC-based susceptibility

		Total number of genomes	Coverage fraction (%)	Sensitivity (%)	PPV (%)	NPV (%)	ME (%)	VME (%)
CRO-first antibiotic strategy (CI 95%)	Ceftriaxone 1 g I.M.	13,633	97.46	100.0 (99.97–100)	99.97 (99.92–99.99)	100.0% (98.91–100.0%)	0.0 (0.0–0.03)	1.14 (0.44–2.89)
	Azithromycin 2 g p.o.	347	74.06	98.08 (95.60–99.18)	99.61 (97.83–99.93)	94.44 (87.65–97.60)	1.92 (0.82–4.40)	1.16 (0.21–6.30)
	Spectinomycin 2 g I.M.	13	100.0	100.0 (77.19–100.0	100.0 (77.19–100.0)	N/A	0.0 (0.0–22.81)	None
	No treatment^a^	77 XDR alert isolates were not predicted for any treatment (spectinomycin MIC data lacking).						
Dual-therapy strategy (CI 95%)	Ceftriaxone 1 g I.M. plus azithromycin 2 g p.o.	13,633	83.61	92.31 (91.82–92.77)	99.70 (99.58–99.79)	57.61 (55.55–59.64)	7.69 (7.23–8.18)	2.57 (1.89–3.58)
	Ceftriaxone 1 g I.M.	2234	84.47	100.0 (99.80–100.0)	100.0 (99.80–100.0)	100.0 (98.91–100.0)	0.0 (0.0–0.20)	0.0 (0.0–1.10)
	Azithromycin 2 g p.o.	347	74.06	98.08 (95.60–99.18)	99.61 (97.83–99.93)	94.44 (87.65–97.60)	1.92 (0.82–4.41)	1.16 (0.21–6.30)
	Spectinomycin 2 g I.M.	13	100.0	100.0 (77.19–100.0)	100.0 (77.19–100.0)	N/A	0.0 (0.0–22.81)	None
	No treatment^a^	77 XDR alert isolates were not predicted for any treatment (spectinomycin MIC data lacking).						
CIP-first antibiotic stewardship strategy (CI 95%)	Ciprofloxacin 500 mg p.o.	11,741	35.30	99.54 (99.29–99.71)	99.69 (99.46–99.82)	99.75 (99.61–99.84)	0.46 (0.29–0.71)	0.17 (0.10–0.29)
	Ceftriaxone 1 g I.M.	9489	96.34	100.0 (99.96–100.0)	99.97 (99.90–99.99)	100.0 (98.90–100.0)	0.0 (0.0–0.04)	0.86 (0.29–2.49)
	Azithromycin 2 g p.o.	347	74.06	98.08 (95.60–99.18)	99.61 (97.83–99.93)	94.44 (87.65–97.60)	1.92 (0.82–4.40)	1.16 (0.21–6.30)
	Spectinomycin 2 g I.M.	13	100.0	100.0 (77.19–100.0)	100.0 (77.19–100.0)	N/A	0.0 (0.0–22.81)	None
	No treatment^a^	77 XDR alert isolates were not predicted for any treatment (spectinomycin MIC data lacking).						
Novel antimicrobials	Zoliflodacin 3 g p.o.	77^a^	100.0	100.0 (95.25–100)	100.0 (95.25–100)	N/A	0.04 (0.0–4.75)	None
	Gepotidacin 3 g + 3 g p.o.	75^a^	97.4	100.0 (95.13–100)	100.0 (95.13–100)	N/A	0.0 (0.0–0.49)	None

Metrics shown are coverage fraction, sensitivity, PPV, NPV, ME and VME for each sequential step. Because SensiTreat only recommends regimens when isolates are predicted to be susceptible, specificity-based metrics are not reported. All 77 XDR alert isolates had MIC data for zoliflodacin and 75 XDR alert isolates had MIC data for gepotidacin, proving susceptibility, accordingly SensiTreat recommended either of these antimicrobials for treatment of the XDR alert cases. Source data are provided as a Source Data file.

*PPV* positive predictive value, *NPV* negative predictive value, *ME* major error, *VME* very major error, *CRO* ceftriaxone, *CIP* ciprofloxacin, *I.M.* intramuscular, *p.o.* oral administration, *MIC* minimum inhibitory concentration, *N/A* not applicable.

^a^Isolates lacked spectinomycin MIC data.

Three independent treatment prioritization strategies were evaluated. Using a susceptibility-guided treatment order in accordance with most current international gonorrhoea management guidelines^2,21^, 13,286 of 13,633 isolates could be assigned ceftriaxone 1 g, corresponding to a coverage fraction of 97.5% and a sensitivity of 100.0% (CI 95%: 99.97–100.0%). The PPV was 99.97% (CI 95%: 99.92–99.99%) and the NPV 100.0% (CI 95%: 98.91–100.0%), which are reported here because they directly reflect the correctness of SensiTreat recommendations and non-recommendations, rather than being dependent on specificity. There were no MEs; however, the VME rate was 1.1% (CI 95%: 0.4–2.9%), corresponding to four misclassifications of ceftriaxone susceptibility. The second susceptibility-guided treatment in the order, azithromycin 2 g monotherapy, was assigned to 257 of the remaining 347 evaluable isolates (coverage fraction 74.1%). Sensitivity was 98.1% (95% CI: 95.6–99.2%), with a PPV of 99.6% (95% CI: 97.8–99.9%) and an NPV of 94.4% (95% CI: 87.6–97.6%). Five azithromycin-susceptible isolates were not assigned azithromycin (ME rate 1.9%, 95% CI: 0.8–4.4%), while one azithromycin-resistant isolate was incorrectly assigned azithromycin, corresponding to a VME rate of 1.2% (95% CI: 0.2–6.3%). Spectinomycin 2 g monotherapy was subsequently assigned to 13 isolates with full concordance (coverage fraction 100%, sensitivity 100% (95% CI: 77.2–100%)). Seventy-seven isolates received no recommendation (spectinomycin MIC data were lacking) and were designated as XDR alert isolates for expert follow-up, including test-of-cure, and comprehensive contact tracing. This reflected data availability rather than prediction failure, i.e., of 90 isolates predicted non-susceptible to both ceftriaxone and azithromycin, phenotypic susceptibility data for spectinomycin were available for only 13 of these isolates, precluding evaluation of spectinomycin-based regimens in the remaining cases. SensiTyper nevertheless recommended spectinomycin regimens for all 77 isolates, but these recommendations could not be assessed due to the absence of corresponding spectinomycin MIC data. All 77 XDR alert isolates had phenotypic MIC data available for zoliflodacin, and 75 of them had phenotypic MIC data for gepotidacin. When zoliflodacin and gepotidacin were included as additional treatment options, all these XDR isolates were susceptible based on available zoliflodacin and gepotidacin MICs and also correctly predicted as susceptible based on the absence of AMR determinants for both these antimicrobials and would have been treatable, with no very major errors observed (Table 3). Finally, SensiTreat recommended ceftriaxone 1 g monotherapy for all NAAT-derived *N. gonorrhoeae* genomes (*n* = 68), showing 100% concordance with matched cultured isolates (58 isolates from 50 samples), while 18 NAAT samples lacked a corresponding isolate.

In settings where ceftriaxone-azithromycin dual therapy remains in use, the difference compared to the strategy described above is simply that isolates are first evaluated jointly for ceftriaxone-azithromycin susceptibility, resulting in a single initial assignment step with a coverage fraction of 83.6%; however, this step retained high performance, with sensitivity 92.3% (95% CI 91.8–92.8%), PPV 99.7% (95% CI 99.6–99.8%), and low ME and VME rates (ME 7.7%, 95% CI 7.2–8.2%; VME 2.6%, 95% CI 1.9–3.6%). Isolates progressing to subsequent single-agent evaluations showed near-perfect performance, with ceftriaxone monotherapy achieving 100% sensitivity and PPV (both 95% CI 99.8–100.0%) and azithromycin achieving sensitivity 98.1% (95% CI 95.6–99.2%) and PPV 99.6% (95% CI 97.8–99.9%), with similar ME and VME rates as the strategy initiated with ceftriaxone 1 g monotherapy (Table 3).

To illustrate antimicrobial stewardship, SensiTreat was additionally applied with ciprofloxacin as the first option. Consequently, 4144 of 11,741 isolates (coverage fraction 35.3%) were directly assigned ciprofloxacin single-dose treatment with high performance (sensitivity 99.5%, CI 95%: 99.3–99.7%; PPV 99.7%, CI 95%: 99.5–99.8%; NPV 99.8%, CI 95%: 99.6–99.8%). The ME rate was 0.5% (CI 95%: 0.3–0.7%), reflecting 19 ciprofloxacin-susceptible isolates not assigned despite being treatable, and the VME rate was 0.2% (CI 95%: 0.1–0.3%), corresponding to 13 ciprofloxacin-resistant isolates incorrectly predicted as ciprofloxacin susceptible. The second treatment in the order, ceftriaxone single-dose monotherapy, was assigned to 9142 isolates with better performance (sensitivity 100%, CI 95%: 99.96–100.0%; PPV 99.97, CI 95%: 99.90–100.0%; NPV 100.0%, CI 95%: 98.9–100.0%). The ME rate was 0.0 (CI 95%: 0.0–0.04%), and the VME rate was 0.9% (CI 95%: 0.3–2.5%), corresponding to three ceftriaxone-resistant isolates incorrectly predicted as ceftriaxone susceptible. Azithromycin single-dose monotherapy, the next option in the order, was assigned to 257 of the 347 remaining isolates with adequate performance (sensitivity 98.1%, CI 95%: 95.6–99.2%; PPV 99.6%, CI 95%: 97.8–99.9%; NPV 94.4%, CI 95%: 87.7–97.6%). The ME rate was 1.9% (CI 95%: 0.8–4.4%) and the VME rate was 1.2% (CI 95%: 0.2–6.3%). Spectinomycin monotherapy was assigned to 13 of the remaining isolates with complete concordance (coverage fraction 1.00; sensitivity 1.00; PPV 1.00; no errors). Regardless of the strategy applied, 77 isolates were classified as XDR alerts because they lacked phenotypic spectinomycin MIC data. Consequently, these isolates were treatable only with zoliflodacin and gepotidacin, for which SensiTyper demonstrated high sensitivity (100%), PPV (100%), coverage (97.4–100%), and low ME rate (0–0.04%) (Table 3).

**Fig. 3:**
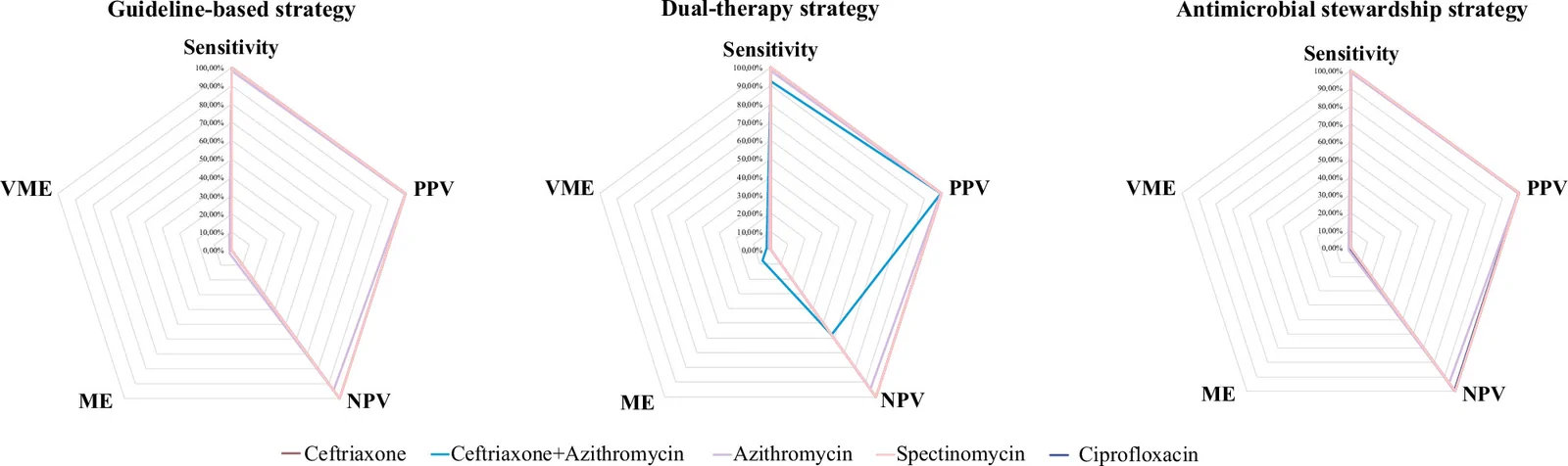
Radar plots of SensiTreat treatment recommendation strategies benchmarked against phenotypic susceptibility for the four antimicrobials with clinical breakpoints (EUCAST v16.0; https://www.eucast.org/bacteria/clinical-breakpoints-and-interpretation/clinical-breakpoint-tables/) that are included in the current gonorrhoea treatment guidelines^2,21^. Three sequential treatment prioritization strategies were evaluated: (i) a susceptibility-guided strategy prioritizing ceftriaxone monotherapy, (ii) a dual-therapy strategy prioritizing ceftriaxone plus azithromycin followed by alternative options, and (iii) an antimicrobial stewardship strategy prioritizing ciprofloxacin when genomic susceptibility was predicted. For each strategy, radar plots summarize sensitivity, positive predictive value (PPV), negative predictive value (NPV), major errors (ME) and very major errors (VME), benchmarked against MIC-based susceptibility testing. Metrics dependent on specificity are not shown, as SensiTreat assigns regimens only to isolates predicted to be susceptible; consequently, the denominator decreases at each sequential treatment step, rendering specificity-based measures less informative. Source data in Table 3.

Overall, treatment orders yielded high concordance between predicted and phenotypic outcomes, with excellent PPV and low error rates across sequential steps. The comparative performance of the three strategies is illustrated in Fig. 3, showing sensitivity, predictive values, ME and VME for each regimen.

## Discussion

We present SensiTyper, a curated rule-based WGS tool that predicts antimicrobial susceptibility in *N. gonorrhoeae* and provides susceptibility-guided individualized treatment recommendations for gonorrhoea. Genomic predictions were analytically evaluated against phenotypic MIC data (current reference standard) from 13,633 temporally and geographically diverse gonococcal strains, including 50 clinical NAAT samples (with 58 matching gonococcal isolates) and 18 clinical NAAT samples with no matching isolate. For ceftriaxone, ciprofloxacin, spectinomycin, and zoliflodacin, SensiTyper showed high sensitivities and specificities, ≥98.9%, and low rates of MEs and especially VMEs. This compares favourably with previous studies using regression-based MIC predictions^45,46^. Furthermore, SensiTyper achieved BAs of >99% for ceftriaxone and ciprofloxacin, and 94.8% for azithromycin, while previous machine-learning approaches^51^ reported lower BAs of 88–92% for ceftriaxone, 93% for ciprofloxacin, and 57–94% for azithromycin across datasets. Azithromycin susceptibility predictions are less consistent, reflecting the polygenic and sometimes subtle mechanisms of decreased azithromycin susceptibility. Importantly, these discrepancies are most pronounced among isolates with azithromycin MICs near the ECOFF value, which are difficult to classify even by phenotypic AST. Nevertheless, the 97.1% specificity of the predictions using SensiTyper ensures that azithromycin-resistant isolates are rarely misclassified as azithromycin susceptible. For gepotidacin, the sensitivity and PPV were 100% and 99.8%, respectively, and no ME were identified in the susceptibility prediction. However, the specificity was 0%, and the VME rate was 100%, which illustrates that gonococcal isolates with low-level phenotypic gepotidacin resistance (MIC = 4 mg/L) using the new US FDA resistance breakpoint (MIC ≥ 4 mg/L; https://www.fda.gov/drugs/development-resources/antibacterial-susceptibility-test-interpretive-criteria) do not contain the only known determinants for gepotidacin resistance in *N. gonorrhoeae* (GyrA A92T plus ParC D86N substitutions)^24,42–44^. Notably, the US FDA breakpoints are AST temporary or provisional interpretative criteria and not yet official clinical breakpoints by the EUCAST and US CLSI. Furthermore, in the microbiologically evaluable population of the gepotidacin phase 3 clinical randomized controlled trial (RCT), no failures to treat urogenital (0/187) and anorectal (0/26) gonorrhoea were detected; however, two (12.5%) of 16 pharyngeal gonorrhoea cases were not cured^24^. Accordingly, no failures (clinical resistance) to cure urogenital or anorectal gonorrhoea using gepotidacin 3 g plus 3 g 10–12 h later have yet been microbiologically confirmed.

The additional strength of SensiTyper is represented by the SensiTreat module, which transforms individual antimicrobial susceptibility predictions for *N. gonorrhoeae* into stepwise, susceptibility-guided treatment recommendations for gonorrhoea. Evaluation of a susceptibility-guided strategy prioritizing ceftriaxone monotherapy as the first-line treatment demonstrated the strongest overall performance among all assessed approaches, and when ceftriaxone resistance was predicted, other treatments were recommended, which reduces the selective pressure and further selection of ceftriaxone resistance. Our results show that the stepwise, susceptibility-guided monotherapy strategy centred on ceftriaxone achieves maximal coverage with minimal risk of inappropriate treatment, while identifying the isolates for which standard options may be insufficient and, e.g., dual-therapy regimens are required. To our knowledge, the predictive performance of dual-therapy regimens has not previously been evaluated, and our results illustrate the potential of SensiTreat to assess treatment combinations directly. Notably, SensiTreat never recommends azithromycin as an independent first-line recommendation, i.e., only after exclusion of ceftriaxone and in exceptional cases when azithromycin susceptibility is predicted with high confidence. For 77 (0.56%) isolates, no treatment was assigned (all were lacking spectinomycin MIC data, so this antimicrobial could not be recommended), and these were automatically flagged as XDR alerts, and expert management, follow-up, including test-of-cure, and comprehensive contact tracing were recommended. However, when zoliflodacin and gepotidacin were included (because they are soon available for treatment in many countries), all 77 XDR isolates were predicted susceptible to both these antimicrobials, and no isolates remained without a treatment option. SensiTreat recommends zoliflodacin and gepotidacin only when susceptibility has been confirmed, which will reduce the selective pressures for the emergence of resistance and preserve their effectiveness. An antimicrobial stewardship-oriented configuration, in which ciprofloxacin was prioritized as a susceptibility-guided first-line treatment, further demonstrated the flexibility of SensiTreat. In this configuration, 35.3% of isolates were immediately assigned ciprofloxacin with adequate performance, and subsequent steps recommending ceftriaxone-based or spectinomycin-based regimens maintained high accuracy. Notably, wild-type GyrA S91 in *N. gonorrhoeae* has been shown to effectively predict susceptibility to ciprofloxacin^55^, and a prospective clinical validation study using a *gyrA*-based PCR confirmed the value of resistance-guided therapy with ciprofloxacin in the US, which can reduce the administration of first-line treatment (ceftriaxone with or without azithromycin)^56^. However, when the ciprofloxacin resistance level is exceedingly high internationally (>90% of countries in the global WHO surveillance report >50% resistance^4^), we need prediction of susceptibility/resistance to also additional antimicrobials and resistance/susceptibility-guided therapy also for these antimicrobials. SensiTyper also incorporates an internal safeguard against uncharacterized or ambiguous genomic backgrounds, requiring detection of the wild-type allele at key loci before antimicrobial susceptibility is assigned. This ensures that novel or previously undetected variants are treated conservatively as non-susceptible, reducing the risk of false-susceptible calls when emerging AMR determinants are not yet defined. Nevertheless, extra caution is warranted in SensiTreat for extragenital gonorrhoea. For example, spectinomycin 2 g monotherapy should not be used when pharyngeal infection has not been excluded, given its limited efficacy at this anatomical site, in such situations, combination therapy with azithromycin 2 g remains advisable^2,21^. Furthermore, zoliflodacin (3 g monotherapy) and gepotidacin (3 g plus 3 g, 10–12 h apart) appear to also have a suboptimal efficacy in the treatment of pharyngeal gonorrhoea (more data are required)^22–24^; both these novel antimicrobials received US FDA approval for treatment of uncomplicated urogenital gonorrhoea^25^. Thus, it is essential that genomic AMR predictions and treatment recommendations also consider the anatomical site and pharmacokinetics/pharmacodynamics in the interpretation and/or reporting of treatment recommendations, and such consideration is included in the SensiTreat reports. Notably, several additional antimicrobials, e.g., gentamicin, delafloxacin, solithromycin, aztreonam, and fosfomycin, have shown a suboptimal cure rate in the treatment of pharyngeal gonorrhoea and failed in their clinical phase 2 or phase 3 RCTs^57–61^.

Notably, SensiTyper (including SensiTreat) is implemented in our clinical routine diagnostic workflow, which has confirmed its feasibility and usefulness, and SensiTyper can be valuable to similarly implement in many other clinical routine diagnostic laboratories, i.e., to predict antimicrobial susceptibility and, in the near future, to also guide individualized treatment of patients with gonorrhoea. For prompt guiding of individualized gonorrhoea treatment, which will provide the full utility and value of SensiTyper, WGS for AMR prediction in NAAT samples will also have to be implemented (because most gonorrhoea patients in many settings are diagnosed using NAATs). Based on the rapid developments in this field (especially with less technically demanding, rapid real-time long-read multiplexed sequencing using, for example, Oxford Nanopore Technology sequencing), real-time WGS sufficient for accurate gonococcal AMR prediction directly from clinical NAAT samples will likely soon be feasible to implement in many clinical routine diagnostic laboratories, that is, when throughput and turnaround times have further improved, costs been reduced and appropriate infrastructure been established. The individualized treatment of gonorrhoea patients, based on accurate AMR prediction, will limit the use of empiric treatment and/or syndromic management, which will reduce the selective pressure and emergence of further AMR. However, SensiTyper can also be valuable for strengthened public health surveillance, i.e., including AMR prediction for AMR surveillance (examining gonococcal isolates and NAAT samples) locally, nationally and internationally, and for providing data for evidence-based population-level treatment policy development. This will effectively supplement the culture-based AMR surveillance, including WGS, where feasible, which should also be further strengthened, because it is technically less challenging, less costly, and can identify and verify new AMR determinants. Notably, SensiTyper is freely available, open source, time-efficient, and quality-controlled, and SensiTyper accurately predicts antimicrobial susceptibility and guides individualized treatment of gonorrhoea patients without the requirements of a comprehensive IT infrastructure and bioinformatic skills. The SensiTyper only requires a conventional laptop (with Linux environment) and internet access to be run, and, as an example, complete AMR prediction and susceptibility-guided treatment recommendations are obtained in approximately 1.5–2 min for 10 genomes with an average genome depth of 50–100×.

The limitations of this study included that sex, gender and age of the gonorrhoea patients were not available. Furthermore, despite that isolates from 68 countries were examined (Supplementary Data, Supplementary Fig. 1), a large proportion of the isolates were from the WHO European Region and comparatively fewer from other WHO regions (enhanced gonococcal WGS is imperative in many WHO regions), causing a suboptimal global representativeness. Relying on MIC-tested cultured isolates can also further skew the dataset, but this was necessary to be able to evaluate the antimicrobial susceptibility prediction. Nevertheless, all known AMR determinants for all examined antimicrobials were represented. Regarding antimicrobial susceptibility prediction, this remains incomplete for azithromycin, reflecting the multifactorial genetic basis of decreased susceptibility and resistance to this drug, and it cannot be excluded that novel resistance determinants for zoliflodacin and gepotidacin, especially, but not exclusively, may emerge. Importantly, SensiTyper infers antimicrobial susceptibility only when wild-type alleles for AMR determinants are explicitly detected, thereby avoiding false-susceptible calls for novel or previously undetected variants, and SensiTyper can easily be refined and updated as new AMR breakpoints, AMR determinants or pharmacokinetic/pharmacodynamic data emerge. It is also important to realize that the correlations between in vitro resistance and clinical cure using EUCAST or other clinical breakpoints are suboptimal for several antimicrobials. For example, infections caused by most ceftriaxone-resistant strains with MICs of 0.25–0.5 mg/L, especially urogenital and anorectal infection, can be effectively cured with ceftriaxone high-dose (1 g) therapy^12,13,62^. A more regular update of EUCAST and other clinical breakpoints is imperative, e.g., when doses of treatment are increased, or new AMR determinants or pharmacokinetic/pharmacodynamic data emerge. Clinical outcome data were not available in this study; accordingly, a prospective, well-designed clinical validation study of the SensiTyper tool, linking AMR prediction, treatment recommendations, and outcome, would be valuable. Furthermore, regulatory approvals for the use of SensiTyper in clinical routine diagnostics in accredited laboratories can be required in some settings. Notably, cefixime, penicillin and tetracycline were not included because they are no longer recommended for gonorrhoea treatment in most countries and the correlations between their chromosomal AMR determinants, MICs and treatment outcomes are suboptimal. Finally, phenotypic MIC data were missing for subsets of isolates and antimicrobials, particularly for spectinomycin, zoliflodacin, and gepotidacin, and nearly all isolates were susceptible to these antimicrobials, resulting in wide confidence intervals for the specificity, NPV and VME rates in the susceptibility prediction.

In conclusion, SensiTyper demonstrates that a curated, rule-based framework based on detection of antimicrobial susceptibility in *N. gonorrhoeae* can bridge the gap between genomic surveillance and clinical decision-making for treatment of gonorrhoea. By combining near-perfect prediction of susceptibility to key antimicrobials with the SensiTreat module’s ability to recommend sequential, susceptibility-guided, individualized treatment regimens, SensiTyper provides interpretable and clinically relevant outputs that can be readily adapted as treatment guidelines evolve. The high accuracy achieved in our analytical validation across a large and temporally and geographically diverse dataset, together with built-in safeguards for emerging, novel or undetected variants, highlights the robustness of this approach. While ongoing refinement will be required to address complex AMR mechanisms such as those influencing azithromycin, these findings show that transparent, knowledge-driven genomic tools can support precision medicine and antimicrobial stewardship efforts in the management of AMR *N. gonorrhoeae*.

## Methods

### Datasets, genome collections and antimicrobial susceptibility testing

*N. gonorrhoeae* genomes with corresponding antimicrobial MIC data were compiled from 32 previous studies (Supplementary Data, Supplementary Fig. 1). A total of 13,133 genomes were downloaded from 68 countries, for which corresponding *N. gonorrhoeae* isolates were collected from 1928 to 2024. All isolates had MIC data for ceftriaxone and azithromycin, while 11,173 (85.1%), 843 (6.4%), 3231 (24.6%), and 2937 (22.4%) had MIC data for ciprofloxacin, spectinomycin, zoliflodacin, and gepotidacin, respectively. Additionally, 568 clinical *N. gonorrhoeae* isolates collected from October 2017 to October 2024 in Sweden were examined. These clinical *N. gonorrhoeae* isolates from Sweden were included to evaluate the feasibility and usefulness of SensiTyper implemented in our clinical routine diagnostic workflow, including gonococcal culture, promptly followed by WGS and analysis with SensiTyper. The MICs (mg/L) of ceftriaxone, azithromycin, ciprofloxacin, and spectinomycin in these isolates were determined by Etest (bioMérieux, Marcy-l'Étoile, France), according to the manufacturer’s instructions. *N. gonorrhoeae* MICs were interpreted according to the EUCAST clinical breakpoints v16.0 or the ECOFF value (MIC > 1 mg/L) for azithromycin (https://www.eucast.org/bacteria/clinical-breakpoints-and-interpretation/clinical-breakpoint-tables/).

### Sequencing of clinical Swedish *Neisseria gonorrhoeae* isolates in a routine diagnostic laboratory

All 568 clinical *N. gonorrhoeae* isolates were cultured on GCAGP^63^ for 18–20 h at 37 °C in a humidified 5% CO_2_-enriched atmosphere, and one single colony was subcultured under the same conditions prior to DNA extraction. Genomic DNA was extracted using the QIASymphony DSP virus/pathogen kit (Qiagen GmbH, Hilden, Germany), according to the manufacturer’s instructions, including RNase treatment. DNA libraries were prepared using the Illumina DNA Prep (Illumina, San Diego, CA, USA) on the Microlab STAR (Hamilton, Reno, NV, USA) liquid handling platform. Libraries were normalized^64^ and sequenced using the MiSeq Reagent Kit v2.

### Bioinformatic analysis

All sequences (*n* = 13,701) were assembled using Spades (v4.0)^65^ with the --careful option. All assemblies were species confirmed using speciator (https://github.com/pathogenwatch-oss/speciator) hosted on the PathogenWatch platform^66^. Furthermore, a core genome multi-locus sequence typing (cgMLST) scheme consisting of 1700 core and 362 accessory genes^38^, was used to characterize all genomes in Ridom Seqsphere+ (v.10.0.1)^67^. A distance matrix from pairwise comparisons of the cgMLST alleles (*n*  =  2062) was generated and a neighbour-joining tree was constructed in Ridom Seqsphere+ (v10.0.1)^67^. The phylogenetic tree was visualized in Microreact^68^.

### Sensityper rule-based logic

**Fig. 4:**
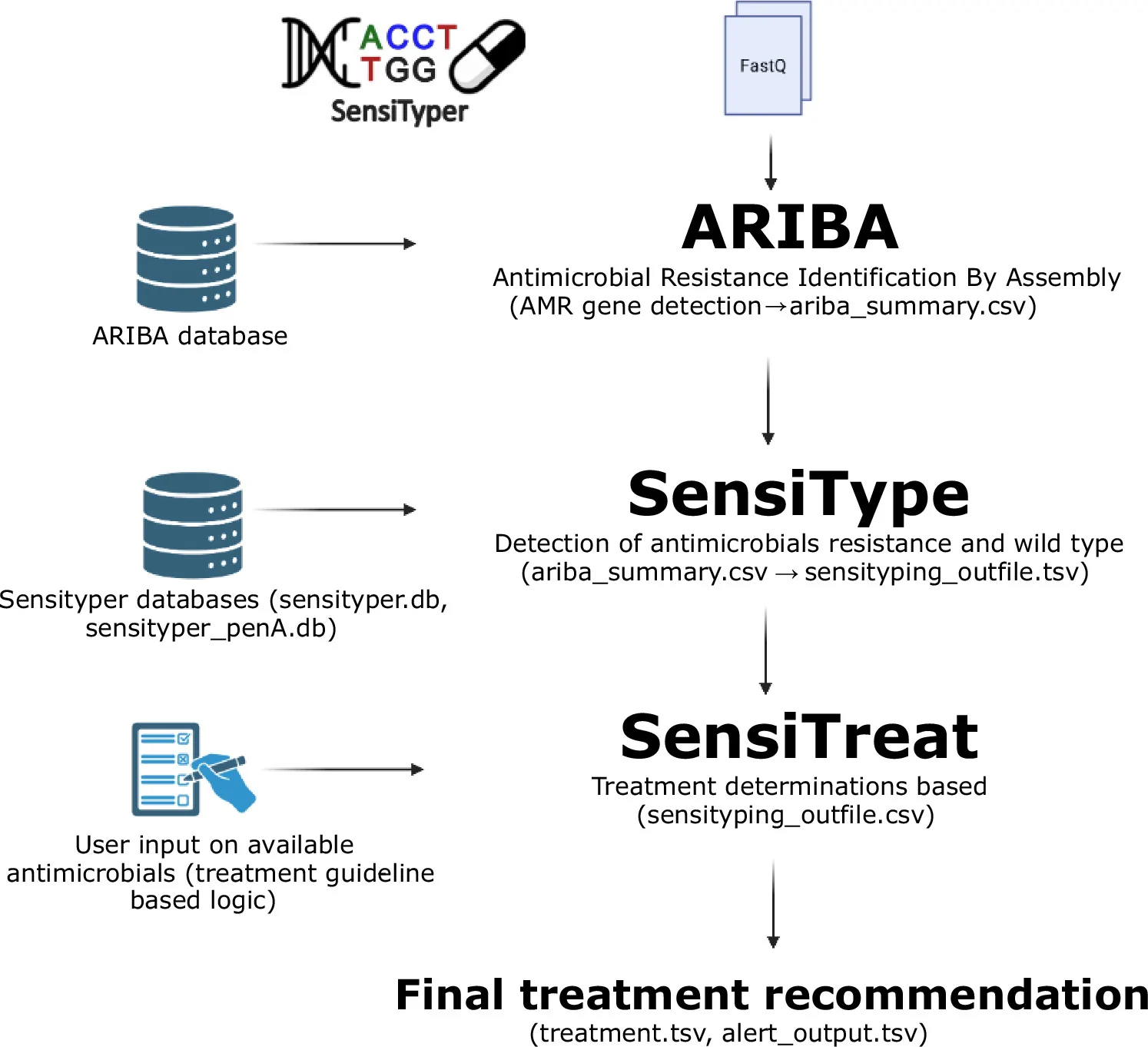
Workflow of the SensiTyper pipeline for genomic prediction of antimicrobial susceptibility in *Neisseria gonorrhoeae* and subsequent susceptibility-guided treatment recommendations for gonorrhoea. Paired-end sequencing reads are analysed with ARIBA^54^ against curated reference databases of AMR determinants. The sensitype module applies antimicrobial-specific rules to interpret detected mutations, mosaic alleles, and structural variants, classifying each isolate as susceptible to ceftriaxone, azithromycin, ciprofloxacin, spectinomycin, zoliflodacin, and gepotidacin. Predicted susceptibility profiles are then integrated by the sensitreat module to generate individualized treatment recommendations (treatment_output.tsv), in which the user defines the prioritization of available antimicrobials. Extensively drug-resistant isolates are flagged (alert_output.tsv). Created in BioRender. Golparian, D. (2026) https://BioRender.com/bay86bq.

SensiTyper is implemented as a modular pipeline with five subcommands: rename, ariba, sensitype, sensitreat, and pipeline (Fig. 4). Each module can be run independently or in series, allowing the pipeline to be adapted to different datasets and user needs.

SensiTyper provides rule-based prediction of antimicrobial susceptibility in *N. gonorrhoeae* and susceptibility-guided treatment recommendations for gonorrhoea using genomic data as input. Figure 4 provides an overview of the SensiTyper approach. Briefly, paired-end reads are used to identify AMR determinants by matching them against a curated reference database containing genes, mutations, and structural variants associated with AMR or antimicrobial susceptibility using ARIBA^54^. For each antimicrobial, the detected determinants are evaluated using antimicrobial-specific decision rules that incorporate single-nucleotide substitutions, gene disruptions, and mosaic alleles. These rules were derived from established AMR determinants and mechanisms reported in the literature and international surveillance studies^2,9,23,24,26–44^. The outcome of the evaluation is a binary classification for each antimicrobial, susceptible or non-susceptible, together with the list of AMR determinants identified. In a second step, the user provides the list of antimicrobials available in their setting and the order of which of these should be prioritized for treatment. Results are integrated across antimicrobials to generate a treatment recommendation, prioritizing susceptibility-guided first-line treatment regimens in the geographic setting, while also considering alternative options when appropriate. A detailed report is generated for all isolate genomes studied, including detection of AMR determinants or wild-type alleles, susceptibility predictions for each drug, and overall recommended susceptibility-guided therapy, with comments for pharyngeal infections when spectinomycin, zoliflodacin or gepotidacin are recommended (due to the suboptimal treatment efficacy for pharyngeal infections)^21–24^ and alerts for XDR cases that require expert management, follow-up, including test-of-cure, and comprehensive contact tracing in a separate output file.

### Obtaining data

SensiTyper requires paired-end sequencing reads processed through ARIBA^54^ as input to generate tables of identified AMR determinants. ARIBA^54^ identifies genetic features by mapping reads against a curated reference database, assembles clusters to call alleles and variants and summarizes them with the summary module. ARIBA can be run separately, but can also be run from SensiTyper in the pipeline mode. For SensiTyping, two complementary reference databases are employed: (i) a general database covering all relevant AMR determinants, including described mosaic/semi-mosaic MtrD alleles associated with increased azithromycin MICs, and corresponding wild types (sensiscript.db), and (ii) a specialized *penA* database (sensiscript.pena.db) designed to distinguish mosaic alleles from wild-type variants. These databases are available through https://github.com/leosanbu/sensityper and contain key genes, mutations, and structural variants associated with AMR in *N gonorrhoeae*, including both presence/absence determinants (e.g., PBP2 alterations, mosaic MtrD) and specific nucleotide substitutions at critical sites (e.g., GyrA S91F, 23S rRNA A2059G or 23S rRNA C2611T)^2,9,23,24,26–44^.

### Preparing input data and detection of genetic markers

Before analysis, raw FASTQ files must be standardized to ensure the correct pairing of forward and reverse reads. The rename module scans user-specified directories, detects common sequencing file suffixes (e.g., *_1.fastq.gz, *_R1.fastq.gz, *_R1_001.fastq.gz), and renames them into a uniform convention (*_R1.fastq.gz/*_R2.fastq.gz). This ensures that downstream steps automatically recognize paired-end reads. For example, FASTQ files in a directory can be standardized with:


sensityper rename --directories input_dir


If users wish to preview the changes without renaming files, the --pre option can be added.

After filenames are standardized, AMR determinants are identified directly from paired-end reads using ARIBA^54^, which compares them against a curated reference database (https://github.com/leosanbu/sensityper). SensiTyper includes an ARIBA wrapper that automates this process across multiple directories, retries failed runs, and generates a combined summary of detected AMR determinants. This step is invoked as:


sensityper ariba --input_dirs dir1,dir2 --output_dir ariba_out --db_path /path/to/ariba_database


This generates per-genome reports (report_new.tsv) and an aggregated ARIBA summary table (ariba_summary.csv), which serve as input to the subsequent SensiTyper modules.

### Rule-based AMR prediction

The ARIBA summary table (ariba_summary.csv) is processed by the sensitype module, which applies antimicrobial-specific decision rules derived from established AMR determinants in *N. gonorrhoeae* (Fig. 4).

The rule set accounts for single-nucleotide polymorphisms (SNPs), indels, gene disruptions, and plasmid-mediated AMR determinants, and produces a binary classification (susceptible or non-susceptible) for each antimicrobial according to alterations in Table 4. The module is run with:

**Table 4:** Antimicrobial resistance determinants detected by SensiTyper^2,9,23,24,26–44^

Antibiotic	Gene	Wild-type position	Known substitution	Functional effect	Wild-type detection
Ceftriaxone	PBP2	Ala311	Val	Ceftriaxone resistance	Yes
	PBP2	Val316	Thr, Pro		Yes
	PBP2	Ala501	Pro		Yes
Azithromycin	23S rRNA	A2059	Guanine	Azithromycin resistance	Yes
	23S rRNA	C2611	Thymine		Yes
	MtrD	N/A	Semi-mosaic and mosaic structure		No
	MtrC	GC dinucleotide deletion in repeat	N/A	Restores azithromycin susceptibility in the presence of MtrD alterations.	No
Ciprofloxacin	GyrA	Ser91	Phe	Ciprofloxacin resistance	Yes
Spectinomycin	16S rRNA	C1192	Thymine	Spectinomycin resistance	Yes
	*rpsE* (S5)	Thr22	Pro		Yes
Zoliflodacin	GyrB	Asp429	Asn, Ala, Val	Zoliflodacin resistance	Yes
	GyrB	Lys450	Thr		Yes
Gepotidacin	GyrA	Ala92	Thr^a^	Gepotidacin resistance	Yes
	ParC	Asp86	Asn^a^		Yes

For each antimicrobial, the key genetic loci involved in antimicrobial resistance, wild-type amino acid or nucleotide positions, and known alterations associated with antimicrobial resistance or altered susceptibility are listed. The table also indicates whether the algorithm detects wild-type alleles.

*PBP2* penicillin-binding protein 2, *N/A* not applicable.

^a^GyrA A92T plus ParC D86N cause high-level gepotidacin resistance^42–44^.


sensityper sensitype --input_AMRtable ariba_summary.csv --sensiscript_outfile sensityping_output.tsv --sensiscript_db sensiscript.db --sensiscript_pena sensiscript.penA.db --sensiscript_antibiotics ceftriaxone,azithromycin,ciprofloxacin,spectinomycin


The example above with four antimicrobials outputs a tab-delimited file listing, for each isolate, the predicted susceptibility per antimicrobial and the underlying AMR determinants.

Predicted susceptibility profiles are then translated into susceptibility-guided treatment recommendations using the sensitreat module and its treatment recommendation logic. This step assigns first-line and alternative regimens, flags XDR isolates, and adds explanatory comments tailored to each isolate to aid interpretation. The module can be executed as


sensityper sensitreat --input_file sensityping_output.tsv --available_antibiotics ceftriaxone,azithromycin,ciprofloxacin,spectinomycin --sensitreat_order ciprofloxacin,ceftriaxone,azithromycin,spectinomycin --alert_output alert_output.tsv --treatment_output treatment_output.tsv


The --sensitreat_order option allows the user to define the relative priority of antimicrobials. For example, if ciprofloxacin is listed first as above, isolates predicted to be susceptible to ciprofloxacin will be assigned this drug for treatment, and ceftriaxone, azithromycin, and spectinomycin will not be considered for any ciprofloxacin-susceptible isolates. In this way, the ordering is critical: it reflects treatment strategies and determines which antimicrobials are selected as first-line and alternative regimens, while also supporting antimicrobial stewardship by allowing the prioritization of agents that are not normally considered first-line therapy for gonorrhoea.

The whole sensityping pipeline can be executed with the pipeline function as below: sensityper pipeline --modules ariba,sensitype,sensitreat --other_arguments "--input_dirs dir1,dir2 --output_dir pipeline_out --db_path /path/to/ariba_db --sensiscript_outfile sensiscript_results.csv --sensitreat_order ciprofloxacin, ceftriaxone, azithromycin, spectinomycin"

In the present study, sensitreat with ceftriaxone monotherapy, a second dual-therapy strategy, and a third strategy with ciprofloxacin as first-line treatment were used to illustrate effective antimicrobial stewardship that could be explored more readily when predicting antimicrobial susceptibility from WGS data.

### Performance evaluation and statistical analysis

We assessed SensiTyper by comparing genomic predictions with phenotypic antimicrobial susceptibility results from the MIC testing and interpretations of the MICs using clinical breakpoints or ECOFF (for azithromycin) from the EUCAST (v16.0; https://www.eucast.org/bacteria/clinical-breakpoints-and-interpretation/clinical-breakpoint-tables/). For zoliflodacin and gepotidacin, the susceptibility breakpoints recently stated by the US FDA were used. For each antimicrobial, predictions were encoded as binary labels and benchmarked against the corresponding binary reference derived from MIC interpretation. Point estimates were calculated for the following metrics: sensitivity, specificity, PPV, NPV, MCC, BA, and FDR. For the definition of XDR, the definition by Tapsall et al. from 2009^69^ was modified (because antimicrobial treatment recommendations and antimicrobials tested in the AST have changed) to align with the contemporary gonococcal population, antimicrobials currently recommended for gonorrhoea treatment, and antimicrobials included in the present AST in most countries. Accordingly, XDR was defined as resistance to ceftriaxone, which translates into resistance to cefixime and azithromycin (i.e., resistance to all antimicrobials currently recommended for treatment, corresponding to category I antimicrobials^69^) plus resistance to ciprofloxacin, which is the only category II or III antimicrobial^69^ that remains tested in many countries.

Analyses were performed in two prespecified comparisons. The primary analysis assessed per-antimicrobial genomic predictions against phenotypic MIC interpretations (SensiTyping vs MIC testing). The secondary analysis evaluated clinical prioritization logic by comparing the pipeline’s selection of first-line or alternative therapy with MIC-based susceptibility results; antibiotics were assessed sequentially according to the user-defined order. All results are reported as point estimates.

Statistical analyses were carried out using the custom Python script sensityping_metrics (https://github.com/leosanbu/sensityper), which computes classification performance for (i) per-antimicrobial susceptibility predictions and (ii) treatment recommendation steps. For per-antibiotic evaluation (--analysis_type predicted_vs_treatment), predicted susceptibility labels (<ABX>_predicted) were compared against the MIC-derived labels (<ABX>_treatment) for each antimicrobial, with “Yes” indicating susceptible/treatable by default. For recommendation-logic evaluation (--analysis_type first_line_vs_treatment), each regimen (including CRO + AZM) was assessed sequentially using <TOKEN>_recommend versus <TOKEN>_treatment. After each step, isolates predicted “Yes” for that token were removed from subsequent steps to reflect the pipeline prioritization logic. In addition to point estimates for sensitivity, specificity, PPV, NPV, BA, and FDR, the script optionally reports uncertainty (95% confidence interval) enabled via --ci_flag.

sensityping_metrics -i metrics_input.tsv -o metrics_etest_vs_treatment.txt -d metrics_out --analysis_type first_line_vs_treatment --order CIP,CRO,AZM + SPC,SPC --radar_flag --radar_antibiotics CIP,CRO,AZM + SPC,SPC --ci_flag --ci_level 0.95

First_line is the predicted first-line treatment from SensiTyper, and treatment is the gold standard susceptibility testing data.

### Reporting summary

Further information on research design is available in the Nature Portfolio Reporting Summary linked to this article.

## Supplementary information

Supplementary Information

Description of Additional Supplementary Information

Supplementary Data 1

Reporting Summary

Transparent Peer Review file

## Source data

Source Data

## Data Availability

All genomic data analysed in the present study have been deposited in international nucleotide archives, and all accession numbers are provided in Supplementary Data. All other data supporting the findings of the present study are available within the paper, including the supplementary material. Source data are provided with this paper.

## Code availability

All code, scripts, tools, and databases are available in our GitHub (https://github.com/leosanbu/sensityper, https://doi.org/10.5281/zenodo.22835141^70^).

## References

[1] World Health Organization (WHO). *WHO Bacterial Priority Pathogens List, 2024: Bacterial Pathogens of Public Health Importance to Guide Research, Development and Strategies to Prevent and Control Antimicrobial Resistance* (World Health Organization (WHO), 2024). https://www.who.int/publications/i/item/97892400934

[2] Unemo, M. et al. Gonorrhoea. *Nat. Rev. Dis. Prim.* **5**, 79 (2019). 10.1038/s41572-019-0128-6 31754194

[3] Maatouk, I. et al. Antimicrobial resistance in *Neisseria gonorrhoeae* in nine sentinel countries within the World Health Organization Enhanced Gonococcal Antimicrobial Surveillance Programme (EGASP), 2023: a retrospective observational study. *Lancet Reg. Health West. Pac.* **61**, 101663 (2025). 10.1016/j.lanwpc.2025.101663 PMC12414356 40922809

[4] Unemo, M. et al. WHO global gonococcal antimicrobial surveillance programmes, 2019–22: a retrospective observational study. *Lancet Microbe* **6**, 101181 (2025). 10.1016/j.lanmic.2025.101181 PMC12715682 41015046

[5] Golparian, D. et al. Antimicrobial-resistant *Neisseria gonorrhoeae* in Europe in 2020 compared with in 2013 and 2018: a retrospective genomic surveillance study. *Lancet Microbe* **5**, e478–e488 (2024). 10.1016/S2666-5247(23)00370-1 38614111

[6] De Silva, D. et al. Whole-genome sequencing to determine transmission of *Neisseria gonorrhoeae*: an observational study. *Lancet Infect. Dis.* **16**, 1295–1303 (2016). 10.1016/S1473-3099(16)30157-8 PMC5086424 27427203

[7] Harris, S. R. et al. Public health surveillance of multidrug-resistant clones of *Neisseria gonorrhoeae* in Europe: a genomic survey. *Lancet Infect. Dis.* **18**, 758–768 (2018). 10.1016/S1473-3099(18)30225-1 PMC6010626 29776807

[8] Sánchez-Busó, L. et al. Europe-wide expansion and eradication of multidrug-resistant *Neisseria gonorrhoeae* lineages: a genomic surveillance study. *Lancet Microbe* **3**, e452–e463 (2022). 10.1016/S2666-5247(22)00044-1 35659907

[9] Golparian, D. et al. Antimicrobial-resistant *Neisseria gonorrhoeae* in eight WHO Enhanced Gonococcal Antimicrobial Surveillance Programme (EGASP) sentinel countries in three WHO regions, 2021–2024: a genomic surveillance study. Preprint at https://ssrn.com/abstract=5839058 (2025). 10.1016/j.ijregi.2025.100624 PMC12008125 40256400

[10] Reimche, J. L. et al. Genomic analysis of 1710 surveillance-based *Neisseria gonorrhoeae* isolates from the USA in 2019 identifies predominant strain types and chromosomal antimicrobial-resistance determinants. *Microb. Genom.* **9**, mgen001006 (2023). 10.1099/mgen.0.001006 PMC10272886 37171855

[11] Miari, V. F., Mosoff, J. M. & Chico, R. M. Minimum inhibitory concentrations of extended-spectrum cephalosporins: a systematic review and meta-analysis of *Neisseria gonorrhoeae* treatment failures. *Sex. Transm. Dis.* **52**, 279–284 (2025). 10.1097/OLQ.0000000000002116 39673499

[12] Fifer, H. et al. Ceftriaxone-resistant *Neisseria gonorrhoeae* detected in England, 2015 to 2024; an observational study. *J. Antimicrob. Chemother.* **79**, 3332–3339 (2023). 10.1093/jac/dkae369 PMC11638718 39417254

[13] Van der Veen, S. Global transmission of the *penA* allele 60.001—containing high-level ceftriaxone-resistant gonococcal FC428 clone and antimicrobial therapy of associated cases: a review. *Infect. Microbe Dis.* **5**, 13–20 (2023).

[14] Unemo, M. et al. World Health Organization Global Gonococcal Antimicrobial Surveillance Program (WHO GASP): review of new data and evidence to inform international collaborative actions and research efforts. *Sex. Health* **16**, 412–425 (2019). 10.1071/SH19023 PMC7035961 31437420

[15] Eyre, D. W. et al. Gonorrhoea treatment failure caused by a *Neisseria gonorrhoeae* strain with combined ceftriaxone and high-level azithromycin resistance, England, February 2018. *Euro Surveill.* **23**, 1800323 (2018). 10.2807/1560-7917.ES.2018.23.27.1800323 PMC6152157 29991383

[16] Pleininger, S. et al. Extensively drug-resistant (XDR) *Neisseria gonorrhoeae* causing possible gonorrhoea treatment failure with ceftriaxone plus azithromycin in Austria, April 2022. *Euro Surveill.* **27**, 2200455 (2022). 10.2807/1560-7917.ES.2022.27.24.2200455 PMC9205165 35713023

[17] Caméléna, F. et al. Emergence of extensively drug-resistant *Neisseria gonorrhoeae*, France, 2023. *Emerg. Infect. Dis.* **30**, 1903–1906 (2024). 10.3201/eid3009.240557 PMC11347006 39084693

[18] Jennison, A. V. et al. Genetic relatedness of ceftriaxone-resistant and high-level azithromycin resistant *Neisseria gonorrhoeae* cases, United Kingdom and Australia, February to April 2018. *Euro Surveill.* **24**, 1900118 (2019). 10.2807/1560-7917.ES.2019.24.8.1900118 PMC6446956 30808445

[19] Ouk, V. et al. High prevalence of ceftriaxone-resistant and XDR *Neisseria gonorrhoeae* in several cities of Cambodia, 2022–23: WHO Enhanced Gonococcal Antimicrobial Surveillance Programme (EGASP). *JAC. Antimicrob. Resist.* **6**, dlae053 (2024). 10.1093/jacamr/dlae053 PMC10993901 38577702

[20] Lan, P. T. et al. The World Health Organization (WHO) Enhanced Gonococcal Antimicrobial Surveillance Programme (EGASP) reports continuously high levels of ceftriaxone resistance across Viet Nam, 2024. *Lancet Reg. Health West. Pac.* **63**, 101709 (2025). 10.1016/j.lanwpc.2025.101709 PMC12547025 41140782

[21] Unemo, M. et al. European guideline for the diagnosis and treatment of gonorrhoea in adults. *Int. J. STD. AIDS* **29**, 956462420949126 (2020). 10.1177/0956462420949126 33121366

[22] Luckey, A. et al. Zoliflodacin versus ceftriaxone plus azithromycin for treatment of uncomplicated urogenital gonorrhoea: an international, randomised, controlled, open-label, phase 3, non-inferiority clinical trial. *Lancet* **407**, 147–160 (2026). 10.1016/S0140-6736(25)01953-1 PMC12784215 41391465

[23] Unemo, M. et al. Microbiological analysis and whole-genome sequencing of *Neisseria gonorrhoeae* from the microbiological failures in the international, zoliflodacin, phase 3, clinical trial for treatment of uncomplicated urogenital gonorrhoea: a retrospective, genomic, observational study. *Lancet Microbe* **7**, 101270 (2026). 10.1016/j.lanmic.2025.101270 41655579

[24] Ross, J. D. C. et al. Oral gepotidacin for the treatment of uncomplicated urogenital gonorrhoea (EAGLE-1): a phase 3 randomised, open-label, non-inferiority, multicentre study. *Lancet* **405**, 1608–1620 (2025). 10.1016/S0140-6736(25)00628-2 40245902

[25] Two new drugs for gonorrhea. *Med. Lett. Drugs Ther.* **68**, 41–43 (2026). 10.58347/tml.2026.1750a 41793382

[26] Unemo, M. & Shafer, W. M. Antimicrobial resistance in *Neisseria gonorrhoeae* in the 21st century: past, evolution, and future. *Clin. Microbiol. Rev.* **27**, 587–613 (2014). 10.1128/CMR.00010-14 PMC4135894 24982323

[27] Tomberg, J., Unemo, M., Ohnishi, M., Davies, C. & Nicholas, R. A. Identification of amino acids conferring high-level resistance to expanded-spectrum cephalosporins in the *penA* gene from *Neisseria gonorrhoeae* strain H041. *Antimicrob. Agents Chemother.* **57**, 3029–3036 (2013). 10.1128/AAC.00093-13 PMC3697319 23587946

[28] Van Hal, S. J., Lahra, M. M. & Whiley, D. M. Determining targets for ceftriaxone resistance detection in *Neisseria gonorrhoeae* based on genotype-phenotype data: an observational study. *Lancet Microbe* **6**, 101199 (2025). 10.1016/j.lanmic.2025.101199 41075810

[29] Galarza, P. G. et al. New mutation in 23S rRNA gene associated with high level of azithromycin resistance in *Neisseria gonorrhoeae*. *Antimicrob. Agents Chemother.* **54**, 1652–1653 (2010). 10.1128/AAC.01506-09 PMC2849369 20123998

[30] Chisholm, S. A., Dave, J. & Ison, C. A. High-level azithromycin resistance occurs in *Neisseria gonorrhoeae* as a result of a single point mutation in the 23S rRNA genes. *Antimicrob. Agents Chemother.* **54**, 3812–3816 (2010). 10.1128/AAC.00309-10 PMC2935028 20585125

[31] Wadsworth, C. B., Arnold, B. J., Sater, M. R. A. & Grad, Y. H. Azithromycin resistance through interspecific acquisition of an epistasis-dependent efflux pump component and transcriptional regulator in *Neisseria gonorrhoeae*. *mBio* **9**, e01419–18 (2018). 10.1128/mBio.01419-18 PMC6083905 30087172

[32] Rouquette-Loughlin, C. E. et al. Mechanistic basis for decreased antimicrobial susceptibility in a clinical isolate of *Neisseria gonorrhoeae* possessing a mosaic-like *mtr* efflux pump locus. *mBio* **9**, e02281–18 (2018). 10.1128/mBio.02281-18 PMC6282211 30482834

[33] Golparian, D. et al. Recent dynamics in *Neisseria gonorrhoeae* genomic epidemiology in Brazil: antimicrobial resistance and genomic lineages in 2017–20 compared to 2015–16. *J. Antimicrob. Chemother.* **79**, 1081–1092 (2024). 10.1093/jac/dkae075 38517452

[34] Ma, K. C. et al. Adaptation to the cervical environment is associated with increased antibiotic susceptibility in *Neisseria gonorrhoeae*. *Nat. Commun.* **11**, 4126 (2020). 10.1038/s41467-020-17980-1 PMC7431566 32807804

[35] Belland, R. J., Morrison, S. G., Ison, C. & Huang, W. M. *Neisseria gonorrhoeae* acquires mutations in analogous regions of *gyrA* and *parC* in fluoroquinolone-resistant isolates. *Mol. Microbiol.* **14**, 371–380 (1994). 10.1111/j.1365-2958.1994.tb01297.x 7830580

[36] Galimand, M., Gerbaud, G. & Courvalin, P. Spectinomycin resistance in *Neisseria* spp. due to mutations in 16S rRNA. *Antimicrob. Agents Chemother.* **44**, 1365–1366 (2000). 10.1128/aac.44.5.1365-1366.2000 PMC89873 10770780

[37] Unemo, M. et al. *Neisseria gonorrhoeae* strain with high-level resistance to spectinomycin due to a novel resistance mechanism (mutated ribosomal protein S5) verified in Norway. *Antimicrob. Agents Chemother.* **57**, 1057–1061 (2013). 10.1128/AAC.01775-12 PMC3553714 23183436

[38] Golparian, D. et al. GyrB in silico mining in 27 151 global gonococcal genomes from 1928–2021 combined with zoliflodacin in vitro testing of 71 international gonococcal isolates with different GyrB, ParC and ParE substitutions confirms high susceptibility. *J. Antimicrob. Chemother.* **78**, 150–154 (2022). 10.1093/jac/dkac366 36308328

[39] Jacobsson, S. et al. High susceptibility to the novel antimicrobial zoliflodacin among *Neisseria gonorrhoeae* isolates in eight WHO Enhanced Gonococcal Antimicrobial Surveillance Programme countries in three WHO regions, 2021–2024. *IJID. Reg.* **15**, 100624 (2025). 10.1016/j.ijregi.2025.100624 PMC12008125 40256400

[40] Alm, R. A. et al. Characterization of the novel DNA gyrase inhibitor AZD0914: low resistance potential and lack of cross-resistance in *Neisseria gonorrhoeae*. *Antimicrob. Agents Chemother.* **59**, 1478–1486 (2015). 10.1128/AAC.04456-14 PMC4325766 25534723

[41] Ahlstrand, J. et al. High in vitro activity of the novel antimicrobial gepotidacin against *Neisseria gonorrhoeae* isolates in eight WHO Enhanced Gonococcal Antimicrobial Surveillance Programme countries in three WHO regions, 2021–24. *J. Antimicrob. Chemother.* **81**, dkaf452 (2026). 10.1093/jac/dkaf452 PMC12802896 41400870

[42] Jacobsson, S., Golparian, D., Scangarella-Oman, N. & Unemo, M. In vitro activity of the novel triazaacenaphthylene gepotidacin (GSK2140944) against MDR *Neisseria gonorrhoeae*. *J. Antimicrob. Chemother.* **73**, 2072–2077 (2018). 10.1093/jac/dky162 PMC6927889 29796611

[43] Scangarella-Oman, N. E. et al. Microbiological analysis from a phase 2 randomized study in adults evaluating single oral doses of gepotidacin in the treatment of uncomplicated urogenital gonorrhea caused by *Neisseria gonorrhoeae*. *Antimicrob. Agents Chemother.* **62**, e01221–18 (2018). 10.1128/AAC.01221-18 PMC6256812 30249694

[44] David, A. et al. In silico gepotidacin target mining among 33 213 global *Neisseria gonorrhoeae* genomes from 1928 to 2023 combined with gepotidacin MIC testing of 22 gonococcal isolates with different GyrA and ParC substitutions. *J. Antimicrob. Chemother.* **79**, 2221–2226 (2024). 10.1093/jac/dkae217 PMC11368423 39004438

[45] Eyre, D. W. et al. WGS to predict antibiotic MICs for *Neisseria gonorrhoeae*. *J. Antimicrob. Chemother.* **72**, 1937–1947 (2017). 10.1093/jac/dkx067 PMC5890716 28333355

[46] Demczuk, W. et al. Equations to predict antimicrobial MICs in *Neisseria gonorrhoeae* using molecular antimicrobial resistance determinants. *Antimicrob. Agents Chemother.* **64**, e02005–e02019 (2020). 10.1128/AAC.02005-19 PMC7038236 31871081

[47] Nakayama, S. et al. New ceftriaxone- and multidrug-resistant *Neisseria gonorrhoeae* strain with a novel mosaic *penA* gene isolated in Japan. *Antimicrob. Agents Chemother.* **60**, 4339–4341 (2016). 10.1128/AAC.00504-16 PMC4914677 27067334

[48] Zhang, C. et al. Emergence, evolution and temporal spread of ceftriaxone-resistant *Neisseria gonorrhoeae* in China from 2002 to 2022: a retrospective genomic surveillance study. *Emerg. Microbes Infect.* **14**, 2579397 (2025). 10.1080/22221751.2025.2579397 PMC12599174 41128429

[49] Lan, P. T. et al. The WHO Enhanced Gonococcal Antimicrobial Surveillance Programme (EGASP) identifies high levels of ceftriaxone resistance across Vietnam, 2023. *Lancet Reg. Health West. Pac.* **48**, 101125 (2024). 10.1016/j.lanwpc.2024.101125 PMC11260586 39040039

[50] Berçot, B. et al. Ceftriaxone-resistant, multidrug-resistant *Neisseria gonorrhoeae* with a novel mosaic *penA-237.001* gene, France, June 2022. *Euro Surveill.* **27**, 2200899 (2022). 10.2807/1560-7917.ES.2022.27.50.2200899 PMC9808317 36695466

[51] Hicks, A. L. et al. Evaluation of parameters affecting performance and reliability of machine learning-based antibiotic susceptibility testing from whole genome sequencing data. *PLoS. Comput. Biol.* **15**, e1007349 (2019). 10.1371/journal.pcbi.1007349 PMC6743791 31479500

[52] Golparian, D., Rapp, E., Hasmats, J. & Unemo, M. Novel approach using automated target enrichment enables culture-independent accurate whole-genome sequencing of *Neisseria gonorrhoeae* directly from clinical urogenital and extragenital specimens. *J. Antimicrob. Chemother.* **80**, 563–566 (2025). 10.1093/jac/dkae446 39680110

[53] Ellington, M. J. et al. The role of whole genome sequencing in antimicrobial susceptibility testing of bacteria: report from the EUCAST Subcommittee. *Clin. Microbiol. Infect.* **23**, 2–22 (2017). 10.1016/j.cmi.2016.11.012 27890457

[54] Hunt, M. et al. ARIBA: rapid antimicrobial resistance genotyping directly from sequencing reads. *Microb. Genom.* **3**, e000131 (2017). 10.1099/mgen.0.000131 PMC5695208 29177089

[55] Allan-Blitz, L. T., Wang, X. & Klausner, J. D. Wild-type gyrase A genotype of *Neisseria gonorrhoeae* predicts in vitro susceptibility to ciprofloxacin: a systematic review of the literature and meta-analysis. *Sex. Transm. Dis.* **44**, 261–265 (2017). 10.1097/OLQ.0000000000000591 PMC5407314 28407640

[56] Klausner, J. D. et al. Resistance-guided treatment of gonorrhea: a prospective clinical study. *Clin. Infect. Dis.* **73**, 298–303 (2021). 10.1093/cid/ciaa596 PMC8282307 32766725

[57] de Vries, H. J. C. et al. Efficacy of ertapenem, gentamicin, fosfomycin, and ceftriaxone for the treatment of anogenital gonorrhoea (NABOGO): a randomised, non-inferiority trial. *Lancet Infect. Dis.* **22**, 706–717 (2022). 10.1016/S1473-3099(21)00625-3 35065063

[58] Hook, E. W. et al. Efficacy and safety of single-dose oral delafloxacin compared with intramuscular ceftriaxone for uncomplicated gonorrhea treatment: an open-label, noninferiority, phase 3, multicenter, randomized study. *Sex. Transm. Dis.* **46**, 279–286 (2019). 10.1097/OLQ.0000000000000971 30985632

[59] Chen, M. Y. et al. Solithromycin versus ceftriaxone plus azithromycin for the treatment of uncomplicated genital gonorrhoea (SOLITAIRE-U): a randomised phase 3 non-inferiority trial. *Lancet Infect. Dis.* **19**, 833–842 (2019). 10.1016/S1473-3099(19)30116-1 31196813

[60] Barbee, L. A., Soge, O. O., Ocbamichael, N., LeClair, A. & Golden, M. R. Single-arm open-label clinical trial of two grams of aztreonam for the treatment of Neisseria gonorrhoeae. *Antimicrob. Agents Chemother.* **65**, e01739–20 (2020). 10.1128/AAC.01739-20 PMC7927842 33077658

[61] Ross, J. D. C. et al. Gentamicin compared with ceftriaxone for the treatment of gonorrhoea (G-ToG): a randomised non-inferiority trial. *Lancet* **393**, 2511–2520 (2019). 10.1016/S0140-6736(18)32817-4 PMC6620599 31056291

[62] Unemo, M. et al. Pharmacodynamic evaluation of ceftriaxone single-dose therapy (0.125-1 g) to eradicate ceftriaxone-susceptible and ceftriaxone-resistant *Neisseria gonorrhoeae* strains in a hollow fibre infection model for gonorrhoea. *J. Antimicrob. Chemother.* **79**, 1006–1013 (2024). 10.1093/jac/dkae063 38497988

[63] Foerster, S. et al. In vitro antimicrobial combination testing of and evolution of resistance to the first-in-class spiropyrimidinetrione zoliflodacin combined with six therapeutically relevant antimicrobials for *Neisseria gonorrhoeae*. *J. Antimicrob. Chemother.* **74**, 3521–3529 (2019). 10.1093/jac/dkz376 31730160

[64] Eyre, D. W., Golparian, D. & Unemo, M. Prediction of minimum inhibitory concentrations of antimicrobials for *Neisseria gonorrhoeae* using whole-genome sequencing. *Methods Mol. Biol.* **1997**, 59–76 (2019). 10.1007/978-1-4939-9496-0_4 31119617

[65] Prjibelski, A., Antipov, D., Meleshko, D., Lapidus, A. & Korobeynikov, A. Using SPAdes De Novo Assembler. *Curr. Protoc. Bioinform.* **70**, e102 (2020). 10.1002/cpbi.102 32559359

[66] Sánchez-Busó, L. et al. A community-driven resource for genomic epidemiology and antimicrobial resistance prediction of *Neisseria gonorrhoeae* at Pathogenwatch. *Genome Med.* **13**, 61 (2021). 10.1186/s13073-021-00858-2 PMC8054416 33875000

[67] Jünemann, S. et al. Updating benchtop sequencing performance comparison. *Nat. Biotechnol.* **31**, 294–296 (2013). 10.1038/nbt.2522 23563421

[68] Argimón, S. et al. Microreact: visualizing and sharing data for genomic epidemiology and phylogeography. *Microb. Genom.* **2**, e000093 (2016). 10.1099/mgen.0.000093 PMC5320705 28348833

[69] Tapsall, J. W., Ndowa, F., Lewis, D. A. & Unemo, M. Meeting the public health challenge of multidrug- and extensively drug-resistant *Neisseria gonorrhoeae*. *Expert Rev. Anti-Infect. Ther.* **7**, 821–834 (2009). 10.1586/eri.09.63 19735224

[70] Golparian, D., Sánchez-Busó, L. & Unemo, M. Genomic surveillance meets clinical practice: rule-based individualized treatment prediction for gonorrhoea using SensiTyper. *Github*10.5281/zenodo.22835141 (2026). PMC13722691 42963361

