# Supplementary material for: Genomic surveillance meets clinical practice: rule-based individualized treatment prediction for gonorrhoea using SensiTyper
Source: Nat Commun. 2026 Oct 30;17:11293. doi: 10.1038/s41467-026-79037-z (PMC13722691; doi:10.1038/s41467-026-79037-z)
Supplement: Supplementary file 1 [file 41467_2026_79037_MOESM1_ESM.pdf]

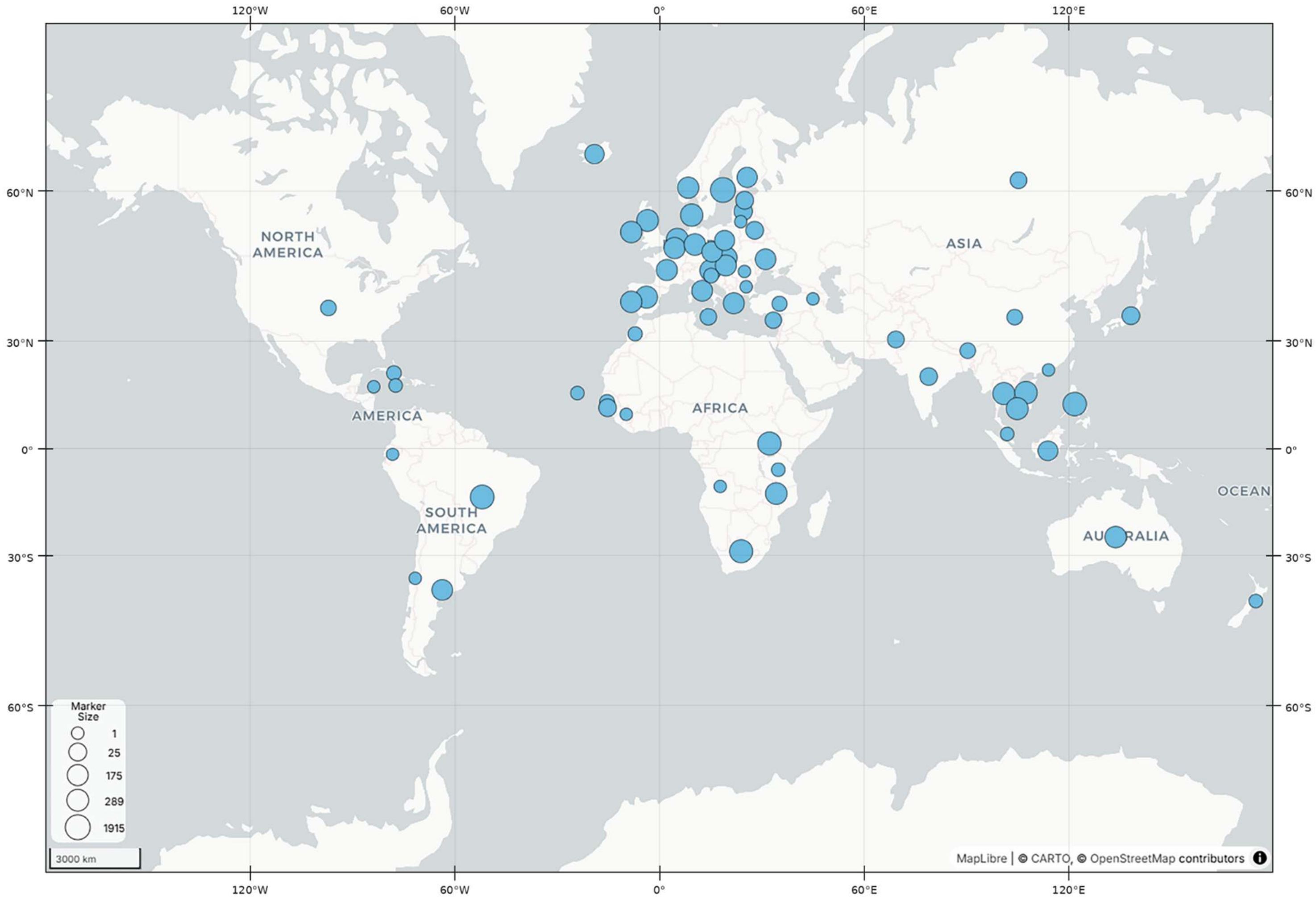

Supplementary Figure 1 | Geographic origin of 13,133 *Neisseria gonorrhoeae* isolates (from 1928-2024) compiled from 32 published studies (Supplementary Table 1), in addition to 568 contemporary clinical isolates (from Sweden, 2017-2024) sequenced in the present study. The dataset covers 68 countries and comprises 13,701 gonococcal genomes. Size of circle is proportional to the number of isolates in each country. Map generated in Microreact (https://microreact.org).
