# Supplementary material for: Genomic surveillance meets clinical practice: rule-based individualized treatment prediction for gonorrhoea using SensiTyper
Source: Nat Commun. 2026 Oct 30;17:11293. doi: 10.1038/s41467-026-79037-z (PMC13722691; doi:10.1038/s41467-026-79037-z)
Supplement: Supplementary file 3 [file 41467_2026_79037_MOESM5_ESM.pdf]

nature portfolio

Peer Review File

Genomic Surveillance Meets Clinical Practice: Rule-Based Individualized Treatment Prediction for Gonorrhoea using SensiTyper

Corresponding Author: Professor Magnus Unemo

This file contains all reviewer reports in order by version, followed by all author rebuttals in order by version.

Version 0:

Reviewer comments:

Reviewer #1

(Remarks to the Author)
Golparian et al. developed a novel SensiTyper, a curated rule-based tool designed to interpret WGS data to predict AST in N. gonorrhoeae and support susceptibility-guided individualized treatment of gonorrhea. In contrast to previous approaches that infer MICs through statistical modeling or machine learning, SensiTyper employs transparent and biologically interpretable rules based on curated genetic determinants of AMR to directly infer susceptibility or AMR categories. Noteworthy, SensiTyper's major strength lies in the SensiTreat module, which converts antimicrobial susceptibility predictions for N. gonorrhoeae into stepwise, susceptibility-guided treatment recommendations. Evaluation of strategies prioritizing ceftriaxone monotherapy as first-line therapy showed the best overall performance among the assessed approaches. Importantly, in SensiTreat, gonorrhea treatments are assigned sequentially, recommending regimens only when susceptibility is predicted and excluding ineffective options until a viable therapy is identified. An antimicrobial stewardship–oriented configuration of the SensiTreat module, prioritizing ciprofloxacin as a susceptibility-guided first-line therapy, illustrates the flexibility of this approach. The inclusion of zoliflodacin approved by the FDA in December 2025, as a conditional downstream option, rather than a universal first-line treatment, is commendable. The authors evaluated SensiTyper using 13,575 N. gonorrhoeae genomes and 50 clinical NAAT samples (with 58 matching gonococcal isolates) and 18 clinical NAAT samples with no matching isolate, collected across diverse temporal and geographic contexts, associated with MIC data, and assessed SensiTyper's predictive performance against established clinical resistance breakpoints or epidemiological cut-off values (ECOFF) for azithromycin. Overall, this is a comprehensive, rigorous, and methodologically sound study with well-presented data and acknowledged limitations, and the manuscript is well-written. I have provided several suggestions for the authors' consideration.
1) Lines 78-91. The authors should consider adding genetic resistance determinants known to confer in vitro resistance to gepotidacin.
2) Lines 136-138. What is the feasibility of WGS directly from clinical NAAT specimens? WGS of N. gonorrhoeae directly from clinical specimens that are positive by NAATs remain technically challenging, primarily because of the low abundance of gonococcal DNA and the high proportion of host and commensal microbial DNA present in such specimens. Although, recent studies have shown that targeted enrichment approaches can substantially improve sequence yield, genome coverage, and depth when sequencing directly from clinical specimens, they have been done in research context with high costs. The manuscript would benefit from a more detailed discussion of the practical feasibility and affordability of implementing such enrichment based approaches in routine surveillance programs. In particular, the authors should consider the cost of enrichment reagents, automation platforms, and sequencing relative to traditional culture based WGS, as well as the scalability of these methods for large scale gonococcal AMR surveillance.
3) Table 1. I commend the authors for providing data on zoliflodacin, one of the two oral antimicrobials approved by the FDA for gonorrhea treatment in December 2026. It would be informative to include published genomes with gepotidacin data.
4) Figure 1. Please make this figure bigger with emphasis on the predicted susceptibilities and susceptibility-guided therapies. The fonts are too small and difficult to read.
5) Although SensiType appears to demonstrate improved performance relative to previously published models for predicting AMR in N. gonorrhoeae, it is well established, and acknowledged by the authors, that the relationship between in vitro susceptibility, as defined by clinical breakpoints, and clinical cure is imperfect for several antimicrobials used in gonorrhea treatment. Prior work has demonstrated that microbiological susceptibility does not always translate directly into clinical cure, particularly for gonococcal infections where pharmacokinetic/pharmacodynamic factors and infection site can influence

outcomes. Consequently, genotype or model based resistance prediction approaches have often required validation against clinical outcomes before being considered for routine clinical implementation. For example, molecular assays predicting ciprofloxacin susceptibility in N. gonorrhoeae based on gyrA mutations were evaluated in prospective clinical studies demonstrating that genotype guided therapy was associated with high cure rates (Clin Infect Dis. 2021 Jul 15;73(2):298-303). While the manuscript acknowledges the absence of clinical outcome or test of cure data as a limitation and notes that treatment recommendations could therefore not be directly correlated with patient outcomes, it does not address whether prospective clinical validation would be necessary before implementing the combined SensiType–SensiTreat approach in clinical practice. Given the recognized limitations of relying solely on in vitro susceptibility as proxies for treatment success, further discussion is warranted regarding why a clinical study evaluating patient outcomes would not be required to substantiate the clinical utility of this novel susceptibility-guided individualized treatment strategy for gonorrhea.
6) Lines 338-340. “All 77 XDR alert isolates were predicted susceptible to zoliflodacin and would have been treatable.” Zoliflodacin is currently approved for the treatment of uncomplicated urogenital gonorrhea, and evidence regarding its efficacy at extragenital sites, particularly the pharynx, remains limited. Pharyngeal gonorrhea has been shown to be associated with higher rates of treatment failure, including with extended spectrum cephalosporins, and is often implicated in cases involving XDR alert isolates. The manuscript would benefit from a more explicit discussion of these limitations, particularly the uncertainty surrounding the effectiveness of zoliflodacin for extragenital infections and the implications for relying on this agent as a treatment option for XDR gonorrhea as predicted in this study due to zoliflodacin susceptibility.
7) Pages 410-440. The rationale for not evaluating the predictive performance of gepotidacin susceptibility in this study is unclear to me. Given the clinical relevance of gepotidacin as a recently FDA-approved treatment for gonorrhea, clarification regarding its exclusion from the analysis would strengthen the manuscript. A brief explanation addressing whether this omission was due to data availability, methodological considerations, or other factors would be informative for readers.
8) Lines 498-503. The discussion would benefit from further elaboration on the need for caution for selection of treatment options for extragenital gonorrhea, citing relevant references. Several studies have documented reduced efficacy of certain antibiotic regimens for extragenital infections, particularly pharyngeal gonorrhea. For example, studies have reported suboptimal outcomes with regimens such as gentamicin monotherapy and aztreonam for pharyngeal gonorrhea. Please clarify that both zoliflodacin and gepotidacin have received FDA approval specifically for the treatment of uncomplicated urogenital gonorrhea, and their efficacy and approval do not currently extend to extragenital infections.

(Remarks on code availability)
Not applicable

Reviewer #2

(Remarks to the Author)
Antimicrobial resistance (AMR) in Neisseria gonorrhoeae is a major public health challenge, and improved approaches for predicting antimicrobial susceptibility are needed. In this manuscript, Golparian and colleagues describe a framework designed to use genomic data to predict susceptibility to multiple antibiotics in N. gonorrhoeae. The authors applied the framework to approximately 13,000 genomes and evaluated its performance under several treatment strategies.

The model demonstrated good performance for ceftriaxone susceptibility prediction, although this finding should be interpreted in the context of the low prevalence of ceftriaxone resistance in the dataset (<3% of isolates). In contrast, performance was substantially lower for azithromycin, with relatively high rates of major errors (7.5%) and very major errors (2.9%). The authors further propose the framework as a tool to inform treatment decision-making, extending beyond prior work that has used PCR- and whole-genome sequencing-based approaches to predict AMR-associated susceptibility.

The manuscript is generally well-written, clearly organized, and addresses an important topic. However, there are several major concerns related to the study's primary contribution and proposed clinical applications. In my view, the central limitation is not the framework itself, which largely builds upon existing approaches for genomic AMR prediction, but rather the lack of a convincing argument about how the proposed tool would improve clinical decision-making relative to current diagnostic and treatment pathways.

Major Comments
1. The clinical relevance and implementation pathway are unclear. The manuscript places substantial emphasis on the potential clinical utility of the proposed model (e.g. lines 435-438, lines 456-458, lines 495-498); however, it remains difficult to envision a realistic implementation pathway within current gonorrhoea diagnostic and treatment workflows.

In high-income settings, most gonococcal infections are diagnosed using nucleic acid amplification tests (NAATs), and treatment is generally provided empirically at the time of diagnosis. In many low-resource settings, which bear a higher burden of AMR, infections are frequently managed syndromically. Consequently, the proposed workflow, which requires bacterial isolation, DNA extraction, and whole-genome sequencing (WGS), does not align well with routine clinical practice in either setting.

Furthermore, the infrastructure requirements, costs, and turnaround times associated with WGS represent substantial barriers to implementation. Because treatment decisions are typically made at the time of diagnosis, it is unclear how the model's predictions would meaningfully influence initial patient management. The authors should provide a more detailed and realistic discussion of the specific clinical scenarios in which this approach would be used and how it would alter patient care relative to current practice.

2. The data presented here have limited clinical incremental value compared with existing approaches. Even in settings where gonococcal cultures are obtained, the clinical value of the proposed approach remains uncertain. When culture is available, phenotypic susceptibility testing can be performed and provides direct measurements of susceptibility that are generally preferred over model-based predictions derived from genomic data. Conversely, laboratories that lack the capacity to perform phenotypic susceptibility testing are unlikely to have routine access to WGS, further narrowing the potential use case for the proposed model.

The manuscript also does not clearly demonstrate that the model provides meaningful advantages over existing molecular resistance prediction approaches. As noted by the authors, prediction of ciprofloxacin susceptibility can be done by rapid targeted PCR assays performed directly on clinical NAAT specimens, providing clinically actionable information without the need for culture and WGS. In contrast, susceptibility prediction for other antibiotics appears to be substantially more complex, as reflected in the authors' performance data, particularly for azithromycin. Given the availability of simpler approaches for ciprofloxacin and the more limited performance observed for other agents, the incremental benefit of a WGS-based susceptibility prediction framework requires stronger justification.

In addition, the authors' own results suggest that a ceftriaxone-first strategy performs reasonably well (Table 3). Because ceftriaxone remains the standard approach in most treatment guidelines, the manuscript should more clearly demonstrate the added clinical value provided by the model beyond current standards of care.

3. The authors appropriately acknowledge in the limitations (Lines 508-512) that in vitro susceptibility does not necessarily correlate with clinical cure. I agree with the decision to frame the model primarily as a susceptibility-prediction tool rather than a predictor of treatment success. However, this distinction further limits the direct clinical applicability of the approach and should be more clearly acknowledged in the discussion.

4. While the manuscript places greater emphasis on “individualized treatment” and clinical decision-making, these applications may be overstated given the limitations discussed above. It may be that the approach is better suited for AMR surveillance and population-level treatment policy development. For example, could the model help characterize regional resistance patterns or inform country-specific treatment recommendations in areas with a high burden of AMR, such as parts of Asia (e.g., Cambodia, China, or Vietnam)? The authors may wish to consider and discuss these potential surveillance applications, which may align more closely with the strengths of the methodology.

Minor Comments:
1. The Introduction is rather long (approximately 4.5 pages) and, at times, reads more like a review article. While the material presented is generally relevant and informative, much of it is not essential for establishing the rationale for the current study. I suggest substantially shortening this section and focusing on the key concepts most relevant to the manuscript, namely the challenges associated with predicting AMR from genetic determinants and the potential for genomic and computational approaches to improve susceptibility prediction. A more concise and focused Introduction would improve the overall readability and impact of the manuscript.

2. Figure 1. Currently, it is overly complex and difficult to follow. It is not immediately clear what the primary message of the figure is or which aspects the reader should focus on. My interpretation is that the figure is intended to illustrate the relationships between resistance-associated mutations and the resulting treatment recommendations; however, the current presentation makes these connections difficult to discern. I encourage the authors to simplify the figure and focus on the key concepts they wish to convey.

3. Methods (lines 541-542): What is the significance of the Sweden isolates? Please explain why they were included.

4. An additional consideration is the regulatory pathway required for implementation of such a tool in clinical practice. In many settings, deployment of a genomic susceptibility prediction tool to guide patient management would require substantial analytical and clinical validation, as well as regulatory approval. These requirements introduce further barriers to implementation and should be considered when discussing the translational potential of the framework.

(Remarks on code availability)
N/A

Reviewer #3

(Remarks to the Author)
In this paper, the authors describe a new tool, SensiTyper, to predict gonococcal AMR. Using a rule-based framework, susceptibility predictions to several antibiotics were described including ceftriaxone, ciprofloxacin and azithromycin. The tool was able to predict susceptibility effectively for four antimicrobials: ceftriaxone, ciprofloxacin, spectinomycin and zoliflodacin.

The article demonstrates how non-machine-learning approaches can provide high accuracy and transparency for AMR prediction, which will be extremely valuable given the increasing AMR reported in N. gonorrhoeae. The tool has the potential to be adapted to other bacterial species and provides a novel approach to the surveillance of AMR. The paper does however, need editing to improve clarity, including altering to improve figures. Finally, the authors should provide adequate supplementary data to make this work reproducible.

Introduction

Line 37/38: repeats "susceptibility-guided individualized treatment recommendations" from line 28/29.
Line 55: "acquired mainly all" does not make sense. Suggest change to "acquired AMR mechanisms to every..."
Line 60: should read "in the most recent decade".
Line 66: should read "guidelines recommend".
Line 114: should read "holds promise".
Line 132-134: This sentence is confusing, please edit.
Generally, the introduction is quite long (4 ½ pages) and should be condensed. There are a few grammatical and typo errors which need proof checking.

Results
Table 1. This summarises antibiotic susceptibility profiles for the collection used and reports number of isolates susceptible to each antimicrobial. Could the authors list in a separate column the number of isolates reported as resistant also? It makes it clearer for the reader to rapidly assess the dataset and proportions of both susceptible and resistant isolates. There is also usually quite a range of MICs reported and it would be useful to list these here.
Line 184, can this be more specific, substantially compared to what?
Line 186, this should be rephrased to: 'Isolates with MICs of <1 mg/L and possessing wildtype GyrB amino acid substitutions D429 and K450 were considered susceptible with all other isolates non-susceptible.' Can the authors state here what the resistant mutations are? I realise these are listed in Table 4 but more detail would be useful here also so the text can be interpreted independently of the table.
The supplementary files should include a table summarising the frequency of isolates by country and by year to show representativeness. The current table does not depict this clearly. As representativeness of the data is mentioned in the paper, this should be better presented in the main results e.g. table, world map. It would also be preferable to have a supplementary excel file or similar listing each isolate, their metadata, MICs (where available) and accession number to ensure reproducibility. The lineages isolates belong to as observed in the phylogenetic analyses should also be listed if possible to also show genomic representativeness.
Line 189: conventionally (e.g. Tapsall et al., 2009) XDR is not defined so narrowly as resistance to just ceftriaxone and azithromycin. This makes the use of this term confusing in the article. Please justify the use of "XDR" in this case or preferably change the term. For example, you could use something like first-line treatment-resistant (FLTR).
Figure 1, Line 198: legend should read "Phylogenetic tree" and should describe what kind of tree it is, and how it was generated. This has been described in the methods but figure legends should also briefly describe this. There is a typo in the figure "stewardship stragety" and the key is missing the orange annotation description for GyrA S91. The key is hard to read, and I suggest it is moved below the tree and the annotation tracks to improve legibility. Overall, the tree makes the annotations hard to read, and many points are too small to be seen.
Figure 2, Line 236: very major error and major error being shown on this plot as 1 – error rate, is confusing for the reader. Please use the true values. Perhaps a table or barplot would be more appropriate than a radar plot to combine all these values in one figure.
Line 280: "susceptible, increased exposure" – reduced susceptibility may make more sense here.
Line 341-343: although the exclusion of cefixime is explained, it seems that it could also be useful in isolates with limited treatment options (Klausner 2022). Similarly, tetracycline is also clinically relevant with the roll out of DoxyPEP in some nations.
Figure 3, Line 389: as above, VME and ME being shown as 1- is confusing on this plot. Please consider alternative methods of displaying these results.

Discussion
Line 415-416: please add this detail to methods too.
Line 442-451: repeating results. Please provide interpretation/synthesis.
Line 527-528: "temporally, geographically and genomically diverse dataset" see earlier note about providing evidence of temporal/geographical distribution. The dataset has an abundance of samples from Europe and North America and comparatively few from Africa or South America. This should be considered in limitations, as should be the fact that relying on MIC tested isolates further skews the dataset. Genomic diversity should also be described rather than just depicted on the phylogeny.
Line 538: Antarctica is not represented, so saying "on 5 continents" would be more accurate.
The discussion is also very long (5 ½ pages). It's difficult to understand what the authors recommend and what the take home message is. Who do the authors plan on the tool being used by? Clinicians? Researchers, public health? What are the next steps for this work? It would be good to have a sense of how long the pipeline takes to run and what computer platform/RAM etc was needed.
My understanding is that SensiTyper works from short read data, as required by ARIBA. Can the tool be adapted to work with genome assemblies for example?

Methods
Line 549: please can the heading be clarified to state this relates to the Swedish collection used in this study.
Please can the authors state why plasmids are not examined in the pipeline.
Table 4. The mutations detected in MtrD should still be listed even though this is due to mosaicism. This is similar to penA where although mosaic alleles confer resistance, a specific set of mutations are known to confer resistance. The authors list the mutations searched for in penA so similarly the ones looked for in MtrD should be listed. This is important as we know other Neisseria species possess mosaic MtrD alleles but these may not all necessarily confer resistance.
Line 564: please reference software where possible: e.g. Ridom Seqsphere+: Jünemann S et al. (2013). Nat Biotechnol. 31:294-6. [PubMed 23563421]
Line 570: please define where can this tool be accessed from/on what infrastructure is it based.

Line 659: tetracycline is still being used to prevent STIs in some settings.

(Remarks on code availability)
Extensive code available that has been well-documented allowing reproducibility

Reviewer #4

(Remarks to the Author)
This is an interesting study that reports a novel tool SensiTyper to classify genomic data of NG isolates according to their drug resistance profile. Assessing drug resistance in NG is notoriously difficult because resistance emerges mostly through combinations of mutations, instead of particular plasmids, mobile elements, or single mutations, as is the case in other bacteria. For this reason, SesiTyper has the potential to be a highly valuable tool.

As I understand it, SensiTyper searches for known antimicrobial resistance mutations/genes in sequencing reads using ARIBA, then applies curated decision rules to classify each isolate as susceptible or resistant for five key antibiotics. Then, the SensiTreat module takes these predictions and applies sequential treatment logic—recommending only susceptible drugs and excluding ineffective options until a viable treatment is found, while flagging extensively drug-resistant (XDR) cases for expert review.

The performance of SensiTyper is remarkable, with over 95% sensitivity and specificity for ceftriaxone and other drugs (e.g. cipro). It does less well for predicting azythronycin resistance, but this is a well known challenge that the authors acknowledge.

I do not have technical concerns but would like to raise a few points before I would recommend this paper for publication:

- It would be good to quantify the extent of bias in the data set used here. While extensive (31 countries). Could the authors comment on how such rules could be updated and what populations should be prioritised (e.g. where is incidence high, but sequencing low)?

- The manuscript mentions that anatomical context matters (i.e. of spectinomycin). However, this is not a variable included in the rules. If this is feasible, I strongly suggest at least expanding the discussion on this point.

- I am a bit concerned about the uncertainty in the specificity of spectinomycin zolfiflodacin, which can be quite low, and probably limits the clinical utility of this tool. If my reading is correct, this would mean that half or more of truly resistant isolates would be incorrectly predicted as susceptible. Could the authors suggest ways to improve such prediction accuracy. From a statistical point, I would think that reporting a posterior probability p(susceptibility+|genotype) = p(susceptibility+)*p(genotype|susceptibility+) / p(genotype). This means that the prior, p(susceptibility), can be drawn from phenotypic data from the population, even in the absence of extensive genomic data.

(Remarks on code availability)
The github repo is neatly organised and well documented.

Reviewer #5

(Remarks to the Author)
I co-reviewed this manuscript with one of the reviewers who provided the listed reports. This is part of the Nature Communications initiative to facilitate training in peer review and to provide appropriate recognition for Early Career Researchers who co-review manuscripts.

(Remarks on code availability)

Version 1:

Reviewer comments:

Reviewer #1

(Remarks to the Author)
In the revised manuscript, the authors have been very responsive to my initial review comments and have made suggested changes that have improved the manuscript. I have no further comments for the authors.

(Remarks on code availability)
Not applicable.

Open Access This Peer Review File is licensed under a Creative Commons Attribution 4.0 International License, which permits use, sharing, adaptation, distribution and reproduction in any medium or format, as long as you give appropriate credit to the original author(s) and the source, provide a link to the Creative Commons license, and indicate if changes were made.
In cases where reviewers are anonymous, credit should be given to 'Anonymous Referee' and the source.
The images or other third party material in this Peer Review File are included in the article's Creative Commons license, unless indicated otherwise in a credit line to the material. If material is not included in the article's Creative Commons license and your intended use is not permitted by statutory regulation or exceeds the permitted use, you will need to obtain permission directly from the copyright holder.
To view a copy of this license, visit https://creativecommons.org/licenses/by/4.0/

Reviewer #1 (Remarks to the Author):
General comments:
"Golparian et al. developed a novel SensiTyper, a curated rule-based tool designed to interpret WGS data to predict AST in N. gonorrhoeae and support susceptibility-guided individualized treatment of gonorrhea. In contrast to previous approaches that infer MICs through statistical modeling or machine learning, SensiTyper employs transparent and biologically interpretable rules based on curated genetic determinants of AMR to directly infer susceptibility or AMR categories. Noteworthy, SensiTyper's major strength lies in the SensiTreat module, which converts antimicrobial susceptibility predictions for N. gonorrhoeae into stepwise, susceptibility-guided treatment recommendations. Evaluation of strategies prioritizing ceftriaxone monotherapy as first-line therapy showed the best overall performance among the assessed approaches. Importantly, in SensiTreat, gonorrhea treatments are assigned sequentially, recommending regimens only when susceptibility is predicted and excluding ineffective options until a viable therapy is identified. An antimicrobial stewardship–oriented configuration of the SensiTreat module, prioritizing ciprofloxacin as a susceptibility-guided first-line therapy, illustrates the flexibility of this approach. The inclusion of zoliflodacin approved by the FDA in December 2025, as a conditional downstream option, rather than a universal first-line treatment, is commendable. The authors evaluated SensiTyper using 13,575 N. gonorrhoeae genomes and 50 clinical NAAT samples (with 58 matching gonococcal isolates) and 18 clinical NAAT samples with no matching isolate, collected across diverse temporal and geographic contexts, associated with MIC data, and assessed SensiTyper's predictive performance against established clinical resistance breakpoints or epidemiological cut-off values (ECOFF) for azithromycin. Overall, this is a comprehensive, rigorous, and methodologically sound study with well-presented data and acknowledged limitations, and the manuscript is well-written. I have provided several suggestions for the authors' consideration."

Authors' response: We are very grateful for the positive response regarding our submitted manuscript. Regarding the provided suggestions, see our point-by-point replies below. Many thanks for these suggestions, which have improved our manuscript.

Specific comments:
1) Lines 78-91. The authors should consider adding genetic resistance determinants known to confer in vitro resistance to gepotidacin.

Authors' response: Revised as suggested. Accordingly, in the revised manuscript, we have added gepotidacin and examinations of also gepotidacin. We have used the breakpoints recently stated by the US FDA (S, MIC≤1 mg/L and R, MIC≥4 mg/L; https://www.fda.gov/drugs/development-resources/antibacterial-susceptibility-test-interpretive-criteria) and in the genomic predictions, we have included that concomitant GyrA A92T and ParC D86N substitutions cause high-level gepotidacin resistance, including appropriate references. Unfortunately, using the resistance breakpoint recently stated by the US FDA (i.e., the only existing gepotidacin resistance breakpoint for N.

gonorrhoeae), low-level gepotidacin resistance (MIC=4 mg/L) does not correlate with presence of the only known gepotidacin resistance determinants in N. gonorrhoeae (GyrA A92T plus ParC D86N). Accordingly, all genomic prediction of gepotidacin susceptibility in N. gonorrhoeae has a low specificity and high VME rate. Nevertheless, this resistance breakpoint might be changed in the future and additional mutations causing resistance to gepotidacin might be identified (which is why we did not include gepotidacin initially, i.e., not sufficiently clear regarding AMR determinants and their correlations to in vitro MICs and treatment outcome). However, this is as good as it can be done at present date and the SensiTyper can later be easily and quickly updated.

2) Lines 136-138. What is the feasibility of WGS directly from clinical NAAT specimens? WGS of N. gonorrhoeae directly from clinical specimens that are positive by NAATs remain technically challenging, primarily because of the low abundance of gonococcal DNA and the high proportion of host and commensal microbial DNA present in such specimens. Although, recent studies have shown that targeted enrichment approaches can substantially improve sequence yield, genome coverage, and depth when sequencing directly from clinical specimens, they have been done in research context with high costs. The manuscript would benefit from a more detailed discussion of the practical feasibility and affordability of implementing such enrichment based approaches in routine surveillance programs. In particular, the authors should consider the cost of enrichment reagents, automation platforms, and sequencing relative to traditional culture based WGS, as well as the scalability of these methods for large scale gonococcal AMR surveillance.

Authors' response: We agree with the Reviewer that this remains challenging. As mentioned by the Reviewer, it is feasible to perform full WGS directly from clinical NAAT specimens. However, unfortunately for appropriate quality and success rate of this type of WGS, target enrichment approaches remain required and these approaches are technically challenging, exceedingly costly, have a limited throughput and require appropriate infrastructure. However, a lot of work to further reduce the cost and increase the speed and throughput is ongoing and full WGS is not necessary for AMR prediction (only appropriate coverage of the AMR determinants). In the Discussion section of the revised manuscript, we have included that WGS for detection of AMR determinants directly from NAATs samples "……. remains in rapid development. Hopefully, real-time WGS for gonococcal AMR prediction directly from clinical NAAT samples will soon be implemented in clinical routine diagnostics to inform treatment, i.e., when throughput and turnaround times have further improved, costs been reduced and appropriate infrastructure has been established.". We think it is essential to strongly emphasize these issues. Moreover, in the Discussion section of the revised manuscript, we have further discussed and stressed these important issues and also mentioned that traditional culture-based AMR surveillance including WGS should not be replaced and instead further implemented internationally, is technically less challenging and cheaper, and finally that our tool (SensiTyper including SensiTreat) can be used for analysis of sequencing reads from both sequenced NAAT

samples and cultured isolates (i.e., also in gonococcal culture-based AMR surveillance programmes). Revised as requested.

3) Table 1. I commend the authors for providing data on zoliflodacin, one of the two oral antimicrobials approved by the FDA for gonorrhea treatment in December 2026. It would be informative to include published genomes with gepotidacin data.

Authors' response: Included as requested. See also our reply to comment #1 above.

4) Figure 1. Please make this figure bigger with emphasis on the predicted susceptibilities and susceptibility-guided therapies. The fonts are too small and difficult to read.

Authors' response: Revised as requested. Accordingly, in the revised manuscript Figure 1 is bigger, including larger fonts. Furthermore, based on comments by Reviewer #2 we have simplified Figure 1 and excluded the individualised treatment recommendations (and the associated large key for these).

5) Although SensiType appears to demonstrate improved performance relative to previously published models for predicting AMR in N. gonorrhoeae, it is well established, and acknowledged by the authors, that the relationship between in vitro susceptibility, as defined by clinical breakpoints, and clinical cure is imperfect for several antimicrobials used in gonorrhea treatment. Prior work has demonstrated that microbiological susceptibility does not always translate directly into clinical cure, particularly for gonococcal infections where pharmacokinetic/pharmacodynamic factors and infection site can influence outcomes. Consequently, genotype or model based resistance prediction approaches have often required validation against clinical outcomes before being considered for routine clinical implementation. For example, molecular assays predicting ciprofloxacin susceptibility in N. gonorrhoeae based on gyrA mutations were evaluated in prospective clinical studies demonstrating that genotype guided therapy was associated with high cure rates (Clin Infect Dis. 2021 Jul 15;73(2):298-303). While the manuscript acknowledges the absence of clinical outcome or test of cure data as a limitation and notes that treatment recommendations could therefore not be directly correlated with patient outcomes, it does not address whether prospective clinical validation would be necessary before implementing the combined SensiType–SensiTreat approach in clinical practice. Given the recognized limitations of relying solely on in vitro susceptibility as proxies for treatment success, further discussion is warranted regarding why a clinical study evaluating patient outcomes would not be required to substantiate the clinical utility of this novel susceptibility-guided individualized treatment strategy for gonorrhea.

Authors' response: We totally agree with the Reviewer. However, antimicrobial susceptibility that we are predicting has a higher correlation with clinical cure than prediction of AMR (and exclusion of the resistant isolates). Nevertheless, regarding the lack of ideal correlation between microbiological in vitro susceptibility and clinical cure, we

have in the revised manuscript included: i) that breakpoints from EUCAST and other organisations stating breakpoints need to be regularly updated when new data become available (increased treatment dose, new AMR determinants and/or new PK/PD data), ii) clearly mentioned the suboptimal cure rate with spectinomycin, zoliflodacin, and gepotidacin (and named several antimicrobials that failed in their phase 2 and/or phase 3 clinical RCTs) in the treatment of pharyngeal gonorrhoea (and also included a comment regarding this issue in the report with treatment recommendations for these antimicrobials), iii) mentioned that considerations regarding pharmacokinetic/pharmacodynamic and anatomical sites are essential when predicting clinical AMR and reporting treatment recommendations, and iv) included that the treatment using ceftriaxone 1 g cures most isolates with ceftriaxone resistance determinants and in vitro ceftriaxone resistance. We totally agree with the Reviewer that a well-designed prospective clinical validation study, linking AMR prediction, treatment recommendations, and clinical treatment outcome, would be important before broad implementation of the SensiTyper-SensiTreat workflow in routine clinical practice. This has been mentioned in the limitation section of the revised manuscript. Revised as requested.

6) Lines 338-340. "All 77 XDR alert isolates were predicted susceptible to zoliflodacin and would have been treatable."
Zoliflodacin is currently approved for the treatment of uncomplicated urogenital gonorrhea, and evidence regarding its efficacy at extragenital sites, particularly the pharynx, remains limited. Pharyngeal gonorrhea has been shown to be associated with higher rates of treatment failure, including with extended spectrum cephalosporins, and is often implicated in cases involving XDR alert isolates. The manuscript would benefit from a more explicit discussion of these limitations, particularly the uncertainty surrounding the effectiveness of zoliflodacin for extragenital infections and the implications for relying on this agent as a treatment option for XDR gonorrhea as predicted in this study due to zoliflodacin susceptibility.

Authors' response: In general, all new antimicrobials for the treatment of gonorrhoea are receiving US FDA approvals for uncomplicated urogenital gonorrhoea, which are due to many issues including too small sample sizes for extragenital infections, too low positivity rate in culture of extragenital samples (and culture traditionally remains the primary endpoint), etc., i.e. not only the cure rate in the clinical phase 3 RCTs. Accordingly, the initial US FDA approval of zoliflodacin (and also gepotidacin) only for uncomplicated urogenital gonorrhoea is in line with the previous approvals of other drugs for the treatment of gonorrhoea (despite that these antimicrobials are used for treatment of both urogenital and extragenital gonorrhoea). However, we totally agree with the Reviewer that the cure rate of zoliflodacin (and gepotidacin) for pharyngeal gonorrhoea does not seem to be ideal, that is, based on the limited evidence currently available (more data are required). In fact, many of the previously and currently used antimicrobials have a suboptimal efficacy in the treatment of pharyngeal gonorrhoea (especially spectinomycin). In the revised manuscript, we have mentioned that zoliflodacin and gepotidacin have received US FDA approval for the treatment of uncomplicated urogenital gonorrhoea (with a reference), that the cure rates for zoliflodacin and gepotidacin (as for spectinomycin) in the treatment of pharyngeal infections were suboptimal in their clinical phase 3 RCTs (with references), and also that additional

evidence regarding treatment with zoliflodacin and gepotidacin of pharyngeal infections are required. Furthermore, we have included comments in the SensiTreat recommendations, i.e., that zoliflodacin, gepotidacin and spectinomycin have a suboptimal efficacy against pharyngeal infections. We have in the SensiTreat reports also included alerts for XDR cases and mentioned that these cases require expert management, follow-up, including test-of-cure, and comprehensive contact tracing. Finally, we have mentioned that genomic treatment recommendations should be interpreted as well as reported considering also the anatomical site and pharmacokinetics/pharmacodynamics. Revised as requested.

7) Pages 410-440. The rationale for not evaluating the predictive performance of gepotidacin susceptibility in this study is unclear to me. Given the clinical relevance of gepotidacin as a recently FDA-approved treatment for gonorrhea, clarification regarding its exclusion from the analysis would strengthen the manuscript. A brief explanation addressing whether this omission was due to data availability, methodological considerations, or other factors would be informative for readers.

Authors' response: We agree with the Reviewer and in the revised manuscript, we have added gepotidacin and examinations of also gepotidacin. See our reply to comment #1 above, which further clarifies how we have included gepotidacin (breakpoints, AMR determinants etc.) and the limitations and reasons to not include gepotidacin initially. Revised as requested.

8) Lines 498-503. The discussion would benefit from further elaboration on the need for caution for selection of treatment options for extragenital gonorrhea, citing relevant references. Several studies have documented reduced efficacy of certain antibiotic regimens for extragenital infections, particularly pharyngeal gonorrhea. For example, studies have reported suboptimal outcomes with regimens such as gentamicin monotherapy and aztreonam for pharyngeal gonorrhea. Please clarify that both zoliflodacin and gepotidacin have received FDA approval specifically for the treatment of uncomplicated urogenital gonorrhea, and their efficacy and approval do not currently extend to extragenital infections.

Authors' response: See our reply to comment #6, Reviewer #1 above. Briefly, in the revised manuscript, we have mentioned that zoliflodacin and gepotidacin have received US FDA approval only for the treatment of uncomplicated urogenital gonorrhoea (with a reference), that the cure rates for zoliflodacin and gepotidacin (as for spectinomycin) in the treatment of pharyngeal infections were suboptimal in their clinical phase 3 RCTs (with references), and also that additional evidence regarding treatment of pharyngeal infections with zoliflodacin and gepotidacin are required. Furthermore, we have included comments in the SensiTreat recommendations, i.e., that zoliflodacin, gepotidacin and spectinomycin have a suboptimal efficacy against pharyngeal infections. We have additionally mentioned that genomic treatment recommendations should be interpreted considering also the anatomical site and pharmacokinetics/pharmacodynamics. Finally, we have added that also several additional antimicrobials, e.g., gentamicin, delafloxacin, solithromycin, aztreonam, and fosfomycin, have shown a suboptimal cure rate in the treatment of pharyngeal

gonorrhoea and failed in clinical phase 2 or phase 3 RCTs (with relevant references). Revised as requested.

Reviewer #1 (Remarks on code availability):
"Not applicable"

Reviewer #2 (Remarks to the Author):
General comments:
"Antimicrobial resistance (AMR) in Neisseria gonorrhoeae is a major public health challenge, and improved approaches for predicting antimicrobial susceptibility are needed. In this manuscript, Golparian and colleagues describe a framework designed to use genomic data to predict susceptibility to multiple antibiotics in N. gonorrhoeae. The authors applied the framework to approximately 13,000 genomes and evaluated its performance under several treatment strategies.

The model demonstrated good performance for ceftriaxone susceptibility prediction, although this finding should be interpreted in the context of the low prevalence of ceftriaxone resistance in the dataset (<3% of isolates). In contrast, performance was substantially lower for azithromycin, with relatively high rates of major errors (7.5%) and very major errors (2.9%). The authors further propose the framework as a tool to inform treatment decision-making, extending beyond prior work that has used PCR- and whole-genome sequencing-based approaches to predict AMR-associated suscesptibility.

The manuscript is generally well-written, clearly organized, and addresses an important topic. However, there are several major concerns related to the study's primary contribution and proposed clinical applications. In my view, the central limitation is not the framework itself, which largely builds upon existing approaches for genomic AMR prediction, but rather the lack of a convincing argument about how the proposed tool would improve clinical decision-making relative to current diagnostic and treatment pathways."

Authors' response: Many thanks for the positive response regarding our submitted manuscript. Regarding the concerns, see our point-by-point replies below. We are grateful for these comments and addressing these comments has improved our manuscript.

Major Comments
1. The clinical relevance and implementation pathway are unclear. The manuscript places substantial emphasis on the potential clinical utility of the proposed model (e.g. lines 435-438, lines 456-458, lines 495-498); however, it remains difficult to envision a realistic implementation pathway within current gonorrhoea diagnostic and treatment workflows.

In high-income settings, most gonococcal infections are diagnosed using nucleic acid amplification tests (NAATs), and treatment is generally provided empirically at the time of diagnosis. In many low-resource settings, which bear a higher burden of AMR, infections are frequently managed syndromically. Consequently, the proposed workflow, which requires

bacterial isolation, DNA extraction, and whole-genome sequencing (WGS), does not align well with routine clinical practice in either setting.

Furthermore, the infrastructure requirements, costs, and turnaround times associated with WGS represent substantial barriers to implementation. Because treatment decisions are typically made at the time of diagnosis, it is unclear how the model's predictions would meaningfully influence initial patient management. The authors should provide a more detailed and realistic discussion of the specific clinical scenarios in which this approach would be used and how it would alter patient care relative to current practice.

Authors' response: We totally understand these concerns of the Reviewer. However, we think that the SensiTyper can be very valuable in many laboratories (clinical and other, e.g., focusing on research, AMR surveillance etc.), i.e., as a time-efficient, effective and quality-controlled workflow for gonococcal AMR prediction and also to guide individualised treatment of gonorrhoea patients in the future (with limited IT infrastructure and bioinformatic skills required for implementation and use). Accordingly, we consider this is an effective solution for the "dry lab component" of gonococcal AMR prediction and future guidance of individualised treatment of patients with gonorrhoea. We also included the 568 clinical N. gonorrhoeae isolates from Sweden that validated SensiTyper implemented in our clinical routine diagnostic workflow, which confirmed the feasibility and usefulness of SensiTyper in our clinical routine diagnostic laboratory. Furthermore, we are continuously using the SensiTyper tool in our clinical routine diagnostic laboratory, which continuously generates additional analytical validation (compared to MIC determination), and we are also reporting the AMR prediction and treatment recommendations to our clinicians. Nevertheless, the "wet lab component" (DNA extraction and WGS of isolates but soon also NAAT samples) needs to further improve for full utility of SensiTyper (especially to guide the treatment of gonorrhoea patients) in the clinical routine diagnostic laboratories. This is also something we and many other work hard on and the progress in this development is rapid. Hopefully, real-time WGS sufficient for gonococcal AMR prediction in also NAAT samples will soon be feasible to implement in many clinical routine diagnostic laboratories, that is, when throughput and turnaround times have further improved, costs been reduced and appropriate infrastructure for this type of WGS has been established. This will give us opportunities to use the AMR prediction to directly inform individualised treatment of patients with gonorrhoea and limit the use of empiric treatment (e.g., treatment using ceftriaxone in ceftriaxone-resistant cases) and/or syndromic management (which would limit the selection pressure and emergence of further resistance). We think that based on the rapid progress in the development of also the "wet lab component", we are getting close to implement this scenario, and when also less technically demanding, rapid real-time long-read multiplexed sequencing using, for example, Oxford Nanopore Technology sequencing can be applied, we can solve the "wet lab component" in both the scenarios the Reviewer mentions, i.e., in both high-income and low-income settings. Nevertheless, it is also important that traditional culture-based AMR surveillance including WGS is not completely replaced and instead further strengthened internationally, i.e., because this remains technically less challenging, cheaper, and most

importantly it is the only way to effectively identify also novel AMR determinants. However, our tool (SensiTyper including SensiTreat) can be effectively used for analysis of the gonococcal WGS also in the culture-based AMR surveillance future (with limited IT infrastructure and bioinformatic skills required for implementation and use). To conclude, in the present study, we cannot present a full solution for the wet lab component, dry lab component, as well as clinical and laboratory infrastructure for the gonococcal AMR prediction and guidance of individualised treatment of patients with gonorrhoea in both high-income settings and low-income settings. However, we present an initial step, i.e., an effective, freely available and simple solution for the dry lab component of the AMR prediction and future guidance of individualised gonorrhoea treatment (to in the future limit empiric treatment and/or syndromic management). Our workflow is also fully open source, can be implemented with limited IT infrastructure and used by many without any sophisticated skills in bioinformatics. In the Introduction and Discussion sections (including limitations) of the revised manuscript, we have further discussed all these important issues. Revised as requested.

2. The data presented here have limited clinical incremental value compared with existing approaches. Even in settings where gonococcal cultures are obtained, the clinical value of the proposed approach remains uncertain. When culture is available, phenotypic susceptibility testing can be performed and provides direct measurements of susceptibility that are generally preferred over model-based predictions derived from genomic data. Conversely, laboratories that lack the capacity to perform phenotypic susceptibility testing are unlikely to have routine access to WGS, further narrowing the potential use case for the proposed model.

The manuscript also does not clearly demonstrate that the model provides meaningful advantages over existing molecular resistance prediction approaches. As noted by the authors, prediction of ciprofloxacin susceptibility can be done by rapid targeted PCR assays performed directly on clinical NAAT specimens, providing clinically actionable information without the need for culture and WGS. In contrast, susceptibility prediction for other antibiotics appears to be substantially more complex, as reflected in the authors' performance data, particularly for azithromycin. Given the availability of simpler approaches for ciprofloxacin and the more limited performance observed for other agents, the incremental benefit of a WGS-based susceptibility prediction framework requires stronger justification.

In addition, the authors' own results suggest that a ceftriaxone-first strategy performs reasonably well (Table 3). Because ceftriaxone remains the standard approach in most treatment guidelines, the manuscript should more clearly demonstrate the added clinical value provided by the model beyond current standards of care.

Authors' response: We consider the SensiTyper and the data presented in the current study are really valuable. Compared to previous studies, we focus on predicting antimicrobial susceptibility which has a higher correlation with both in vitro antimicrobials susceptibility testing and clinical cure than prediction of AMR (and subsequent exclusion of the resistant isolates). The performance characteristics of the SensiTyper (including SensiTreat) were also better than previously reported using other workflows, tools or

models (see discussion about these and references in our revised manuscript). We additionally have included novel AMR determinants (e.g., mosaic/semi-mosaic MtrD associated with increased MICs of azithromycin and penA mutations (indicating specific ceftriaxone-resistance causing mosaic penA alleles) highly associated with ceftriaxone resistance) and also the novel antimicrobials zoliflodacin and gepotidacin. None of these AMR determinants or new antimicrobials were included in previous models and papers. However, as mentioned above, we totally agree that we are only presenting an initial step, i.e., an effective, freely available, and simple solution (requiring limited IT infrastructure and bioinformatic skills) for the dry lab component of the AMR prediction and future guidance of individualised gonorrhoea treatment (to in the future limit empiric treatment and/or syndromic management), and more improvements regarding the wet lab component and clinical infrastructure are required (but the developments of also these are rapidly progressing). Due to the high resistance levels to many antimicrobials and the rapid development of AMR in gonococci, we also think we need prediction of susceptibility/resistance to many antimicrobials, in order to in the future be able to provide individualised treatment of patients with gonorrhoea (also because all antimicrobials are not available in all settings). Regarding ciprofloxacin, the problem is that the resistance is so high internationally (50-60% across Europe, >70% in many Latin American countries, and 90-100% in most African and Asian countries). Furthermore, in many countries (including all European Union countries) there is a warning regarding wide use of fluoroquinolones, i.e., the use is restricted due to the risk of disabling, long-lasting and potentially irreversible side effects, and they should mostly be used if no other alternatives and mostly for very serious infections. Due to these reasons, the rapid PCR assays for detection of ciprofloxacin resistance or ciprofloxacin susceptibility unfortunately have limited usefulness in many international settings (i.e., despite that they are appropriate PCRs and they have been properly evaluated analytically and clinically). Nevertheless, use of ciprofloxacin for treatment of some gonorrhoea cases will reduce the use of first-line treatment (ceftriaxone with or without azithromycin), which is why we have presented also the ciprofloxacin stewardship strategy in the present paper. A similar ciprofloxacin stewardship strategy can also be applied without WGS and instead based on a PCR as the Reviewer mentions, which we have added in the revised manuscript (together with references). Regarding susceptibility prediction for other antimicrobials, it is in most situations only azithromycin that is tricky (and azithromycin use needs to be restricted and reduced anyway), i.e., using the current breakpoints (ECOFFs) stated by EUCAST and CLSI. That is, the performance characteristics can never be ideal because the identical mosaic/semi-mosaic MtrD alleles cause low-level azithromycin resistance in some gonococcal strains, while in other gonococcal strains the azithromycin MIC is increased but still just below the breakpoint for resistance (so they are interpreted as susceptible). Accordingly, the problem is that these AMR determinants have not been considered when stating the resistance breakpoint, which means that you in validations of genomic prediction compared to phenotypic in vitro susceptibility testing always will get discrepancies. Nevertheless, our results were comparatively not too bad, i.e., ≥92% (95% CI: 92.0-93.0%) sensitivity and specificity. Regarding full clinical value of the SensiTyper

(including SensiTreat and its treatment recommendations), this can only be shown in a prospective clinical validation study (and ideally the wet lab component and lab/clinical infrastructure need to be in place before such study is performed). This can hopefully show also the value of avoiding treating resistant cases with incorrect antimicrobials (e.g., treatment using ceftriaxone in ceftriaxone-resistant cases). In the revised manuscript, we have mentioned that a prospective clinical validation study is required. As mentioned above we are also already successfully using the SensiTyper (including SensiTreat) in our clinical setting. We also included in the present study the 568 clinical N. gonorrhoeae isolates from Sweden that validated SensiTyper implemented in our clinical routine diagnostic workflow, which confirmed the feasibility and usefulness of SensiTyper in our clinical routine diagnostic laboratory. Furthermore, we are continuously using the SensiTyper tool in our clinical routine diagnostic laboratory, which continuously generates additional analytical validation (compared to MIC determination), and we are also reporting both the AMR prediction and treatment recommendations to our clinicians. However, as described above it is again important to stress that we are in the present study only presenting an effective, freely available, and simple solution (requiring limited IT infrastructure and bioinformatic skills) for the dry lab component of the AMR prediction and future guidance of individualised gonorrhoea treatment, and more improvements regarding the wet lab component and clinical infrastructure are required (currently in rapid developments) for full clinical utility. In the Introduction section and especially Discussion section (including limitations) of the revised manuscript, we have further discussed all these important issues. See also our reply to comment #1, Reviewer #1 above. Revised as requested.

3. The authors appropriately acknowledge in the limitations (Lines 508-512) that in vitro susceptibility does not necessarily correlate with clinical cure. I agree with the decision to frame the model primarily as a susceptibility-prediction tool rather than a predictor of treatment success. However, this distinction further limits the direct clinical applicability of the approach and should be more clearly acknowledged in the discussion.

Authors' response: We can unfortunately never get around the inherent limitation that in vitro susceptibility does not correlate with clinical cure in all cases and we have now more clearly acknowledged this in the discussion. However, antimicrobial susceptibility that we are predicting has a higher correlation with clinical cure than prediction of AMR (and exclusion of the resistant isolates). Briefly, in the revised manuscript, we have expanded the discussion regarding this (and described as limitations); including mentioning that ceftriaxone 1 g treats most gonococcal infections with in vitro ceftriaxone resistance and ceftriaxone resistance determinants, that the cure rates using spectinomycin, zoliflodacin and gepotidacin in the treatment of pharyngeal gonorrhoea are suboptimal (which we also comment in the treatment recommendations for these antimicrobials), that we need to have the EUCAST and other resistance breakpoints continuously updated and consider also anatomical site and PK/PD, that novel resistance determinants to many antimicrobials, especially the new antimicrobials (zoliflodacin and gepotidacin), will likely emerge, that the SensiTyper can be easily refined and updated as new AMR breakpoints, AMR determinants

or PK/PD data emerge, and that a prospective well-designed clinical validation study of the SensiTyper tool, linking AMR prediction, treatment recommendations and outcome, would be valuable. Despite that the in vitro susceptibility (and accordingly the genomic AMR prediction) does not correlate with clinical cure in all cases, we should not stop performing in vitro susceptibility testing and developing genetic AMR prediction. These are important for individualised treatment (in the ideal scenario) but also for AMR surveillance that is the essential fundament for updating guidelines for empiric treatment and/or syndromic management. Instead, we should try to optimise the in vitro susceptibility testing (incl. breakpoints) and genomic AMR prediction, as well as clearly acknowledge the inherent limitations, i.e., as we have now done in the revised manuscript. Revised as requested. See also our reply to comment #1, Reviewer #1 above.

4. While the manuscript places greater emphasis on "individualized treatment" and clinical decision-making, these applications may be overstated given the limitations discussed above. It may be that the approach is better suited for AMR surveillance and population-level treatment policy development. For example, could the model help characterize regional resistance patterns or inform country-specific treatment recommendations in areas with a high burden of AMR, such as parts of Asia (e.g., Cambodia, China, or Vietnam)? The authors may wish to consider and discuss these potential surveillance applications, which may align more closely with the strengths of the methodology.

Authors' response: See our reply to comment #1, Reviewer #1 above. We totally agree that we are only presenting an initial step, i.e., an effective, freely available, and simple solution (requiring limited IT infrastructure and bioinformatic skills) for the dry lab component of the AMR prediction and future guidance of individualised gonorrhoea treatment (to in the future limit empiric treatment and/or syndromic management), and more improvements regarding the wet lab component and clinical infrastructure are required (but the developments of also these are rapidly progressing). In the Introduction and Discussion sections (including limitations) of the revised manuscript, we have further discussed these important issues, including potential AMR surveillance applications (local, national, and international AMR surveillance based on WGS but also conventional Sanger sequencing or PCRs detecting AMR determinants), including AMR prediction for population-level treatment policy development. Revised as requested.

Minor Comments:

1. The Introduction is rather long (approximately 4.5 pages) and, at times, reads more like a review article. While the material presented is generally relevant and informative, much of it is not essential for establishing the rationale for the current study. I suggest substantially shortening this section and focusing on the key concepts most relevant to the manuscript, namely the challenges associated with predicting AMR from genetic determinants and the potential for genomic and computational approaches to improve susceptibility prediction. A more concise and focused Introduction would improve the overall readability and impact of

the manuscript.

Authors' response: We totally agree with the Reviewer and in the revised manuscript, despite adding some words to address Reviewers' comments, we have substantially shortened (reduced the number of words by 30%) and focused the Introduction section as suggested.

2. Figure 1. Currently, it is overly complex and difficult to follow. It is not immediately clear what the primary message of the figure is or which aspects the reader should focus on. My interpretation is that the figure is intended to illustrate the relationships between resistance-associated mutations and the resulting treatment recommendations; however, the current presentation makes these connections difficult to discern. I encourage the authors to simplify the figure and focus on the key concepts they wish to convey.

Authors' response: Figure 1 aimed to include mainly all data on an individual isolate/case-based level, however, we totally agree with the Reviewer that it became too complex. In the revised manuscript, we have simplified Figure 1 for increased clarity. Accordingly, we have excluded the individualized treatment recommendations (last three annotation tracks and their comprehensive key "Susceptibility-guided strategies"). We have subsequently made the figure, including its fonts, substantially larger. Revised as suggested.

3. Methods (lines 541-542): What is the significance of the Sweden isolates? Please explain why they were included.

Authors' response: These 568 clinical N. gonorrhoeae isolates from Sweden were included to evaluate our clinical routine diagnostic workflow including gonococcal culture, directly followed by WGS, and prompt analysis with the SensiTyper. Accordingly, we wanted to evaluate and confirm that the SensiTyper is feasible as well as useful to implement in our clinical routine diagnostic laboratory. This has been further clarified in the revised manuscript.

4. An additional consideration is the regulatory pathway required for implementation of such a tool in clinical practice. In many settings, deployment of a genomic susceptibility prediction tool to guide patient management would require substantial analytical and clinical validation, as well as regulatory approval. These requirements introduce further barriers to implementation and should be considered when discussing the translational potential of the framework.

Authors' response: We agree with the Reviewer. Regarding analytical validation, we think that the present study provides comprehensive validation (based on examination of nearly 14000 gonococcal genomes, including all known key determinants for resistance to examined antimicrobials, from 68 countries), and we also included the 568 clinical N. gonorrhoeae isolates from Sweden that validated SensiTyper implemented in our clinical

routine diagnostic workflow, which confirmed the feasibility and usefulness of SensiTyper in our clinical routine diagnostic laboratory. Furthermore, we are continuously using the SensiTyper tool in our clinical routine diagnostic laboratory, which continuously generates additional analytical validation (compared to MIC determination). Nevertheless, we need additional validation with more global gonococcal isolates. Regarding clinical validation, we totally agree that a prospective well-designed clinical validation study, including AMR prediction, treatment recommended, treatment given and outcome of the treatments in the patients, is important. Finally, requirements of regulatory approvals, which will differ in different geographic settings, based on comprehensive analytical and clinical validations need to be considered. In the revised manuscript, we have focused the discussion section and included further discussions regarding how the SensiTyper can be beneficially used, clarified that we performed an analytical validation and that further validation is required, i.e., especially a prospective clinical validation study, discussed additional barriers to implementation (e.g., remaining challenges with WGS directly from NAAT samples), and that specific regulatory approvals can be required, especially in accredited laboratories, in some settings. Finally, we have substantially expanded the limitations section, including several of these issues (more global gonococcal strains should be evaluated, clinical outcome data were not available, a prospective clinical validation study would be valuable etc.), in the revised manuscript. Revised as requested.

Reviewer #2 (Remarks on code availability):
"N/A"

"The github repo is neatly organised and well documented."

Authors' response: We are grateful for the positive response regarding the content and organisation of our referenced github.

Reviewer #3 (Remarks to the Author):
General comments:
"In this paper, the authors describe a new tool, SensiTyper, to predict gonococcal AMR. Using a rule-based framework, susceptibility predictions to several antibiotics were described including ceftriaxone, ciprofloxacin and azithromycin. The tool was able to predict susceptibility effectively for four antimicrobials: ceftriaxone, ciprofloxacin, spectinomycin and zoliflodacin.

The article demonstrates how non-machine-learning approaches can provide high accuracy and transparency for AMR prediction, which will be extremely valuable given the increasing AMR reported in N. gonorrhoeae. The tool has the potential to be adapted to other bacterial species and provides a novel approach to the surveillance of AMR. The paper does however, need editing to improve clarity, including altering to improve figures. Finally, the authors should provide adequate supplementary data to make this work reproducible."

Authors' response: We are grateful for the positive response regarding our submitted manuscript. Regarding the editing to improve clarity, see our point-by-point replies to the Reviewer's comments below. Addressing these comments has improved our manuscript. We have also improved the figures in accordance with the comments by all the Reviewers and adequate supplementary data have been provided in the revised manuscript.

Introduction
Line 37/38: repeats "susceptibility-guided individualized treatment recommendations" from line 28/29.

Authors' response: We agree with the Reviewer, however, we think it is adequate to have this mentioned both in the objectives as well as conclusions of our manuscript. We hope this is acceptable for the Reviewer.

Line 55: "acquired mainly all" does not make sense. Suggest change to "acquired AMR mechanisms to every..."

Authors' response: Revised as suggested.

Line 60: should read "in the most recent decade".

Authors' response: Revised as suggested.

Line 66: should read "guidelines recommend".

Authors' response: When shortening the Introduction section based one Reviewer's comments, this sentence was excluded.

Line 114: should read "holds promise".

Authors' response: Revised as suggested.

Line 132-134: This sentence is confusing, please edit.

Authors' response: Revised for clarification as suggested.

Generally, the introduction is quite long (4 ½ pages) and should be condensed. There are a few grammatical and typo errors which need proof checking.

Authors' response: In the revised manuscript, despite adding some words to address Reviewers' comments, the Introduction section has been substantially condensed (reduced the number of words by 30%) and it has also been proof checked as suggested.

Results

Table 1. This summarises antibiotic susceptibility profiles for the collection used and reports number of isolates susceptible to each antimicrobial. Could the authors list in a separate column the number of isolates reported as resistant also? It makes it clearer for the reader to rapidly assess the dataset and proportions of both susceptible and resistant isolates. There is also usually quite a range of MICs reported and it would be useful to list these here.

Authors' response: Revised as suggested. Accordingly, for the six antimicrobials (now including also gepotidacin), the proportion of susceptible isolates, proportion of resistant isolates (despite that our focus is on prediction of susceptibility), and MIC ranges are provided.

Line 184, can this be more specific, substantially compared to what?

Authors' response: Revised for clarification as suggested.

Line 186, this should be rephrased to: 'Isolates with MICs of <1 mg/L and possessing wildtype GyrB amino acid substitutions D429 and K450 were considered susceptible with all other isolates non-susceptible.' Can the authors state here what the resistant mutations are? I realise these are listed in Table 4 but more detail would be useful here also so the text can be interpreted independently of the table.

Authors' response: In the revised manuscript, we are using the susceptibility breakpoints recently stated by the US FDA for zoliflodacin and gepotidacin (https://www.fda.gov/drugs/development-resources/antibacterial-susceptibility-test-interpretive-criteria). Accordingly, this sentence has been revised and we do not need to mention the target amino acids (or any mutations increasing the MICs) for zoliflodacin (or gepotidacin). Revised as suggested.

The supplementary files should include a table summarising the frequency of isolates by country and by year to show representativeness. The current table does not depict this clearly. As representativeness of the data is mentioned in the paper, this should be better presented in the main results e.g. table, world map. It would also be preferable to have a supplementary excel file or similar listing each isolate, their metadata, MICs (where available) and accession number to ensure reproducibility. The lineages isolates belong to as observed in the phylogenetic analyses should also be listed if possible to also show genomic representativeness.

Authors' response: In the revised manuscript, we have mentioned only that the dataset was temporally and geographically diverse, i.e., not anything about its representativeness. Furthermore, we have included among the limitations that despite the examined isolates were from 68 countries (and all known AMR determinants for all examined antimicrobials were represented), a large proportion of the isolates were from WHO European Region and

comparatively fewer from WHO Region of the Americas and WHO African, Southeast Asian, Western Pacific and Eastern Mediterranean regions, causing a suboptimal global representativeness. We have also mentioned that relying only on MIC-tested cultured isolates can further skew the dataset (but this is necessary to rely on to be able to evaluate the antimicrobial susceptibility prediction). Furthermore, we have as requested revised Supplementary Table 1, that is, so it is now describing in detail the number of isolates in each country and year, by study. Moreover, the BioProject accession number (PRJEB number) for each study material is provided. Finally, we have in the Supplementary material included a world map showing all countries (and number of isolates from each country) represented in the presented study. However, when metadata and exact MICs have not been collected systematically (and these are also not available for many isolates and studies where MTAs or DTAs have prohibited uploading these data as isolate-based data) and no lineages have been assigned for the isolates, we do not think it is exceedingly valuable (for the present study) and even feasible to prepare an Excel file listing each of the nearly 14 000 isolates in the present study (which would also not add much additional information compared to what provided in Supplementary Table 1, the world map and provision of the accession numbers). We hope this is acceptable for the Reviewer. Revised as requested (and feasible).

Line 189: conventionally (e.g. Tapsall et al., 2009) XDR is not defined so narrowly as resistance to just ceftriaxone and azithromycin. This makes the use of this term confusing in the article. Please justify the use of "XDR" in this case or preferably change the term. For example, you could use something like first-line treatment-resistant (FLTR).

Authors' response: The conventional definition of MDR and XDR gonococci by Tapsall et al. 2009 is outdated and can no longer be used. This is because antimicrobial treatment recommendations and antimicrobials tested in the AST have changed. Accordingly, we used an XDR definition considering these changes and our XDR definition (based on antimicrobials routinely tested in Euro-GASP, WHO GASP and WHO EGASP (ceftriaxone, cefixime, azithromycin and ciprofloxacin) as well as in many national GASPs) is also used by several European countries and it has been suggested by ECDC. Accordingly, in the present study XDR was defined as resistance to ceftriaxone, which translates into resistance to also cefixime, and azithromycin (i.e., resistance to all antimicrobials currently recommended for treatment, corresponding to category I antimicrobials (Tapsall et al. 2009)) plus resistance to ciprofloxacin, which is the only category II or III antimicrobial (Tapsall et al. 2009) that remains tested in many countries. In the revised manuscript, we have clearly described why we cannot use the conventional XDR definition (Tapsall et al. 200) and also in detail how we defined XDR in the present study (and how our definition aligns with category I-III antimicrobials defined by Tapsall et al. 2009). Currently, there is no internationally agreed definition of XDR N. gonorrhoeae, however, we hope we will soon have this, and the definition then needs to be based on antimicrobials recommended and used in the gonorrhoea treatment as well as antimicrobials included in the AST. We think it is really important to talk about XDR

gonococci and we want to avoid suggesting any new definition such as FLTR, which can confuse the readers too much (and first-line treatment recommended in different countries also differs). We are grateful for this comment and we hope our response and changes in the manuscript are acceptable for the Reviewer.

Figure 1, Line 198: legend should read "Phylogenetic tree" and should describe what kind of tree it is, and how it was generated. This has been described in the methods but figure legends should also briefly describe this. There is a typo in the figure "stewardship stragety" and the key is missing the orange annotation description for GyrA S91. The key is hard to read, and I suggest it is moved below the tree and the annotation tracks to improve legibility. Overall, the tree makes the annotations hard to read, and many points are too small to be seen.

Authors' response: In the revised manuscript, we have changed to Phylogenetic tree and briefly described what kind of tree it is and how it was generated (i.e., as previously described in more detail in the Methods section). Based on several comments by the Reviewers, we have also made this figure less complex by excluding the individualized treatment recommendations (last three annotation tracks and their comprehensive Key: "Susceptibility-guided strategies"). The remaining Key (and all text) has been made larger, moved below the tree and annotation tracks, and the orange annotation description for GyrA S91 has been added. Revised as requested.

Figure 2, Line 236: very major error and major error being shown on this plot as 1 – error rate, is confusing for the reader. Please use the true values. Perhaps a table or barplot would be more appropriate than a radar plot to combine all these values in one figure.

Authors' response: Revised as suggested, i.e., in the revised manuscript we have used the true values for both major and very major errors in Figure 2. All the detailed values are also provided for detailed review and reproducibility in Table 2. Finally, there are so many variables and values, so we consider a radar plot is more appropriate and illustrative than a barplot (we tried this and it became more confusing), especially when we now use the true values for all variables.

Line 280: "susceptible, increased exposure" – reduced susceptibility may make more sense here.

Authors' response: We referred to this as "susceptible, increased exposure" because we use EUCAST breakpoints in our manuscript and this is EUCAST's category for "intermediate susceptibility" or "reduced susceptibility". Anyway, in the revised manuscript we have clarified that this is EUCAST's category and additionally added "(reduced susceptibility)" where we first mention this category. Revised as suggested.

Line 341-343: although the exclusion of cefixime is explained, it seems that it could also be useful in isolates with limited treatment options (Klausner 2022). Similarly, tetracycline is

also clinically relevant with the roll out of DoxyPEP in some nations.

Authors' response: We have purposively avoided to include cefixime due to several reasons. Firstly, cefixime is no longer recommended for treatment of gonorrhoea in most countries (ceftriaxone is clearly a more effective alternative). Secondly, based on available evidence, the use of cefixime in the treatment of gonorrhoea has selected for resistance to cefixime, but also ceftriaxone (due to effective selection of mosaic penA alleles). Thirdly, the correlations between the AMR determinants, MICs and treatment outcomes for cefixime are suboptimal (especially but not exclusively for oropharyngeal infections), which limits the AMR prediction and the SensiTyper and other AMR prediction models will not result in sufficiently high sensitivity and specificity for clinical decisions (i.e., either a high sensitivity or a high specificity has to be compromised). Accordingly, due to these reasons we really prefer to not include cefixime, which may also result in a broader use of cefixime again. Regarding tetracycline/doxycycline, this may show limited efficacy against gonorrhoea in some settings when used as DoxyPEP, however, the resistance to tetracycline/doxycycline is exceedingly high in most countries and we do not think it has potential to be reused in the treatment of diagnosed established gonococcal infections. Furthermore, there are no recommended tetracycline treatment regimen available internationally any longer and, as for cefixime, the correlations between the chromosomal AMR determinants, MICs and treatment outcomes are suboptimal. In the limitations section of the revised manuscript, we have clearly mentioned that we have not included cefixime, penicillin or tetracycline and we have briefly mentioned the reasons for this.

Figure 3, Line 389: as above, VME and ME being shown as 1- is confusing on this plot. Please consider alternative methods of displaying these results.

Authors' response: Revised as suggested, i.e., in the revised manuscript we have used the true values for both major and very major errors also in Figure 3. There are so many variables and values, so we consider a radar plot is more appropriate and illustrative than a barplot (we tried this and it became more confusing), especially when we now use the true values for all variables.

Discussion

Line 415-416: please add this detail to methods too.

Authors' response: As mentioned above, in the limitations section of the revised manuscript we have clearly mentioned that we have not included cefixime (as well as penicillin and tetracycline) and we have also briefly mentioned the reasons for this. Revised as suggested.

Line 442-451: repeating results. Please provide interpretation/synthesis.

Authors' response: Revised as suggested, i.e., avoided repeating too much results without any interpretation/synthesis.

Line 527-528: "temporally, geographically and genomically diverse dataset" see earlier note about providing evidence of temporal/geographical distribution. The dataset has an abundance of samples from Europe and North America and comparatively few from Africa or South America. This should be considered in limitations, as should be the fact that relying on MIC tested isolates further skews the dataset. Genomic diversity should also be described rather than just depicted on the phylogeny.

Authors' response: We agree with the Reviewer. As mentioned above, in the Supplementary material of the revised manuscript, we have provided additional information about the gonococcal isolates examined and their temporal/geographic origin. Furthermore, in the limitations section of the revised manuscript, we have mentioned the suboptimal global representativeness (clearly more quality-assured gonococcal WGS work in especially Africa, South America, Asia and Middle East is essential) and we have also mentioned that we are relying on cultured and MIC tested isolates (which are the only possible to rely on when evaluating antimicrobial susceptibility and/or AMR prediction based on the AMR determinants compared to MICs). Regarding genomic diversity, we agree this is not an ideal word (especially when not defined, not evaluated properly and it is not clear about the characteristics of the genomic diversity ideal for this type of study). Consequently, we have excluded "genomically diverse" in the revised manuscript.

Line 538: Antarctica is not represented, so saying "on 5 continents" would be more accurate.

Authors' response: Correct, and in the revised manuscript we have only kept "from 68 countries".

The discussion is also very long (5 ½ pages). It's difficult to understand what the authors recommend and what the take home message is. Who do the authors plan on the tool being used by? Clinicians? Researchers, public health? What are the next steps for this work? It would be good to have a sense of how long the pipeline takes to run and what computer platform/RAM etc was needed.

Authors' response: We totally agree with the Reviewer. In the revised manuscript, we have substantially revised and focused the discussion section, and more clearly describe where and how SensiTyper can be used, and what the next steps as well as take home messages are. Briefly, we have clarified that we hope the SensiTyper can be implemented in many clinical routine diagnostic laboratories, i.e., for prediction of antimicrobial susceptibility and at least in the future also to directly guide individualised treatment of patients with gonorrhoea. For guiding of individualised treatment of gonorrhoea patients, WGS for AMR prediction in NAAT samples will also have to be implemented (because most gonorrhoea patients in many settings are diagnosed using NAATs). The development of this

type of WGS (rapid DNA extraction, library preparation and WGS) that we and many other work on is rapidly progressing and, hopefully, real-time WGS sufficient for accurate gonococcal AMR prediction directly from clinical NAAT samples will soon be feasible to implement in many clinical routine diagnostic laboratories, that is, when throughput and turnaround times have further improved, costs been reduced and appropriate infrastructure for this type of WGS has been established. The individualised treatment of gonorrhoea patients, based on AMR prediction, is certainly important because it will limit the use of empiric treatment and/or syndromic management (which would limit the selection pressure and emergence of further resistance). However, an appropriate prospective clinical validation study, linking AMR prediction, treatment recommended, given and outcome, and requirements of regulatory approvals, which will differ in different geographic settings, will then have to be considered (especially in accredited laboratories). Notably, in our clinical routine diagnostic laboratory, we have already started to implement the SensiTyper, including AMR prediction and reports of the predicted antimicrobial susceptibility and treatment recommendations that are sent to our clinicians. However, the SensiTyper can also be valuable for public health, i.e., AMR surveillance (examining gonococcal isolates and/or NAAT samples) locally, nationally and internationally, including AMR prediction for population-level treatment policy development. This has also been mentioned in the revised manuscript, together with the fact that traditional culture-based AMR surveillance including WGS should not be replaced or stopped and instead further strengthened internationally, because it is technically less challenging, cheaper, and the only way to identify and verify new AMR determinants. Thus, we think that the SensiTyper can be very valuable in many laboratories (clinical and other, e.g., focusing on research, AMR surveillance etc.), for clinicians receiving the reports of the AMR prediction and treatment recommendations, as well as for public health experts (especially these working with AMR surveillance). SensiTyper is also freely available, open source, time-efficient, effective and quality-controlled and can be used for accurate gonococcal AMR prediction and also to guide individualised treatment of gonorrhoea patients with very limited IT infrastructure and bioinformatic skills. Basically, the SensiTyper only requires a conventional laptop (with Linux environment) and internet access to be used, and a run takes approximately 1.5-2 min per genome (average genome depth of around 50-100x). In the Discussion section (including limitations) of the revised manuscript, we have discussed all these important issues. Unfortunately, because there were many discussions and limitations to add (based on the constructive comments by the Reviewers), we have not managed to shorten the Discussion section (despite excluding a lot of information from the initially submitted manuscript). However, when the Discussion section now is much more focused, we hope this acceptable to the Reviewer.

My understanding is that SensiTyper works from short read data, as required by ARIBA. Can the tool be adapted to work with genome assemblies for example?

Authors' response: The tool can be adapted to work also with genome assemblies, however, we think this would decrease the quality and usefulness of the tool. Accordingly,

by using the sequencing reads (instead of assembly) i) sequencing coverage, depth and quality can be assessed when required, ii) reads can directly after sequencing and QC be examined for AMR determinants, and iii) it can be assessed if all alleles of specific genes are mutated, e.g., how many of the four 23S rRNA gene alleles that have mutations in A2059 or C2611, which are associated with azithromycin resistance (and the azithromycin MIC differs based on the number of mutated 23S rRNA gene alleles).

Methods

Line 549: please can the heading be clarified to state this relates to the Swedish collection used in this study.

Authors' response: Clarified as suggested.

Please can the authors state why plasmids are not examined in the pipeline.

Authors' response: Gonococcal plasmids are only relevant for plasmid-associated resistance to penicillins and tetracyclines. Because we have not included penicillins and tetracyclines in the present study (for our reasons to exclude these, see comment above and limitations section in the revised manuscript), we have not examined plasmids. Furthermore, MICs of penicillins and tetracyclines were not available for the majority of isolates examined, accordingly no AMR predictions could be evaluated.

Table 4. The mutations detected in MtrD should still be listed even though this is due to mosaicism. This is similar to penA where although mosaic alleles confer resistance, a specific set of mutations are known to confer resistance. The authors list the mutations searched for in penA so similarly the ones looked for in MtrD should be listed. This is important as we know other Neisseria species possess mosaic MtrD alleles but these may not all necessarily confer resistance.

Authors' response: In mosaic penA alleles, we and others have identified the amino acid substitutions that are present in all mosaic penA alleles causing resistance in currently spreading ceftriaxone-resistant strains, i.e., PBP2 A311V and/or V316T/P. This is why these can be provided in Table 4. However, we do not yet have similar ideal "indicator amino acid substitutions" for all types of mosaic or semi-mosaic MtrD causing increased MICs of azithromycin. Accordingly, in the SensiTyper all the mosaic/semi-mosaic alleles associated with increased MICs of azithromycin are instead detected using our developed database including all described mosaic/semi-mosaic MtrD associated with increased azithromycin MIC (and this database is regularly updated). Notably, in some earlier publications, amino acid substitutions in R714 and/or K823 were described as the key substitutions associated with increased MICs of macrolides in the most frequently identified mosaic MtrD alleles (Lyu M, Moseng MA, Reimche JL, et al. Cryo-EM Structures of a Gonococcal Multidrug Efflux Pump Illuminate a Mechanism of Drug Recognition and Resistance. mBio 2020; 11: e00996-20. Ma KC, Mortimer TD, Grad YH. Efflux Pump Antibiotic Binding Site Mutations Are Associated with Azithromycin Nonsusceptibility in Clinical Neisseria gonorrhoeae Isolates. mBio 2020; 11: e01509-20). However, since then additional mosaic/semi-mosaic MtrD alleles associated with increased MICs of azithromycin have been identified and this is why the SensiType cannot search only for these amino acid substitutions. For further clarification, in the Methods section of the revised manuscript, we have included: "…a general database covering all relevant AMR determinants, including described mosaic/semi-mosaic MtrD

alleles associated with increased azithromycin MICs, and corresponding wild types (sensiscript.db)".

Line 564: please reference software where possible: e.g. Ridom Seqsphere+: Jünemann S et al. (2013). Nat Biotechnol. 31:294-6. [PubMed 23563421]

Authors' response: Included as suggested.

Line 570: please define where can this tool be accessed from/on what infrastructure is it based.

Authors' response: As mentioned in the "Code availability" section of the revised manuscript, all code, scripts, tools, and databases are publicly available in our github (https://github.com/leosanbu/sensityper).

Line 659: tetracycline is still being used to prevent STIs in some settings.

Authors' response: As mentioned above, tetracycline/doxycycline may show limited efficacy against gonorrhoea in some settings when used as DoxyPEP, however, the resistance to tetracycline/doxycycline is exceedingly high in most countries and we do not think it has potential to be reused in the treatment of diagnosed established gonococcal infections. Furthermore, there are no recommended tetracycline treatment regimen available internationally any longer and the correlations between the chromosomal AMR determinants, MICs and treatment outcomes are suboptimal. In the limitations section of the revised manuscript, we have clearly mentioned that we have not included tetracycline and the reasons for this.

Reviewer #3 (Remarks on code availability):
"Extensive code available that has been well-documented allowing reproducibility."

Authors' response: We are grateful for the positive response regarding our code availability.

Reviewer #4 (Remarks to the Author):
"This is an interesting study that reports a novel tool SensiTyper to classify genomic data of NG isolates according to their drug resistance profile. Assessing drug resistance in NG is notoriously difficult because resistance emerges mostly through combinations of mutations, instead of particular plasmids, mobile elements, or single mutations, as is the case in other bacteria. For this reason, SesiTyper has the potential to be a highly valuable tool.

As I understand it, SensiTyper searches for known antimicrobial resistance mutations/genes in sequencing reads using ARIBA, then applies curated decision rules to classify each isolate as susceptible or resistant for five key antibiotics. Then, the SensiTreat module takes these predictions and applies sequential treatment logic—recommending only susceptible drugs and excluding ineffective options until a viable treatment is found, while flagging extensively drug-resistant (XDR) cases for expert review.

The performance of SensiTyper is remarkable, with over 95% sensitivity and specificity for ceftriaxone and other drugs (e.g. cipro). It does less well for predicting azythronycin resistance, but this is a well known challenge that the authors acknowledge.

I do not have technical concerns but would like to raise a few points before I would recommend this paper for publication:"

Authors' response: Many thanks for the positive response regarding our submitted manuscript. Regarding your few points, we are grateful for these and the Reviewer can see our point-by-point replies below.

"- It would be good to quantify the extent of bias in the data set used here. While extensive (31 countries). Could the authors comment on how such rules could be updated and what populations should be prioritised (e.g. where is incidence high, but sequencing low)?"

Authors' response: We are very sorry but we think it is mainly impossible to appropriately quantify the extent of any bias in our dataset (i.e., beyond what we done in our calculations of the AMR predictions, treatment predictions and analytical validations). However, in the revised manuscript, we have substantially expanded the text regarding limitations. This includes that we lacked global representativeness (but we included isolates from 68 countries and all known key AMR determinants for the examined antimicrobials), and we stress the importance of more gonococcal genomic data linked to validated antimicrobial MICs from additional countries and regions. Furthermore, we have provided additional information about the isolates examined and their origin in the Supplementary information. We have also clarified that the SensiTyper (including SensiTreat) can be easily refined and updated when new AMR breakpoints, AMR determinants or PK/PD data emerge. Finally, we have mentioned that AMR breakpoints from EUCAST and other organisations stating breakpoints also need to regularly be updated when new data emerges (e.g., increased treatment dose, new AMR determinants or new PK/PD data available). In fact, it is much quicker and easier to gather evidence and to refine and update the SensiTyper-SensiTreat tool than the conventional MIC-based breakpoints. Revised as requested.

"- The manuscript mentions that anatomical context matters (i.e. of spectinomycin). However, this is not a variable included in the rules. If this is feasible, I strongly suggest at least expanding the discussion on this point."

Authors' response: Revised as requested. Accordingly, in the revised manuscript, we have mentioned that zoliflodacin and gepotidacin have received US FDA approval only for the treatment of uncomplicated urogenital gonorrhoea (with a reference), that the cure rates for zoliflodacin and gepotidacin (as for spectinomycin) in the treatment of pharyngeal infections were suboptimal in their clinical phase 3 RCTs (with references), and also that additional evidence regarding treatment of pharyngeal infections with zoliflodacin and gepotidacin are required. Furthermore, we have included comments in the SensiTreat

recommendations, i.e., that zoliflodacin, gepotidacin and spectinomycin have a suboptimal efficacy against pharyngeal infections. We have additionally mentioned that genomic treatment recommendations should be interpreted as well as reported considering also the anatomical site and PK/PD. Finally, we have added that also several other antimicrobials, e.g., gentamicin, delafloxacin, solithromycin, aztreonam, and fosfomycin, have shown a suboptimal cure rate in the treatment of pharyngeal gonorrhoea and failed in clinical phase 2 and/or phase 3 RCTs (with relevant references). See also our reply to comment #6, Reviewer #1 above.

"- I am a bit concerned about the uncertainty in the specificity of spectinomycin zolfiflodacin, which can be quite low, and probably limits the clinical utility of this tool. If my reading is correct, this would mean that half or more of truly resistant isolates would be incorrectly predicted as susceptible. Could the authors suggest ways to improve such prediction accuracy. From a statistical point, I would think that reporting a posterior probability p(susceptibility+|genotype) = p(susceptibility+)*p(genotype|susceptibility+) / p(genotype). This means that the prior, p(susceptibility), can be drawn from phenotypic data from the population, even in the absence of extensive genomic data."

Authors' response: We understand the concerns of the Reviewer when reading the wide confidence intervals for the specificity in the susceptibility prediction for spectinomycin and zoliflodacin. However, in fact the specificity in the susceptibility prediction for spectinomycin and zoliflodacin were 100% (no isolates predicted susceptible using the genomic prediction were resistant in the phenotypic MIC determination). However, the lower end of the 95% confidence interval was low (44-56%), which is only because there were so few isolates resistant to these antimicrobials. For example, there were only 3 spectinomycin-resistant isolates (none were incorrectly identified as susceptible), and the specificity was 100% but the estimate was imprecise (i.e., wide confidence interval) because there were so few resistant isolates (i.e., this exceedingly low prevalence makes the confidence interval for the specificity and NPV very wide). Accordingly, this is no inherent problem with the specificity of susceptibility prediction and instead only with the datasets available to validate this type of prediction. Isolates resistant or non-susceptible to spectinomycin and zoliflodacin are exceedingly rare, which is of course good for treatment but not for calculating the 95% CIs for the specificity (and NPV and VME rates) in this case. Regarding posterior probabilities, we would really like to avoid including also this (and hope this is acceptable for the Reviewer). The reason is that we think readers of this paper are more familiar with discussions regarding and understanding of sensitivity, specificity, NPV and PPV (including understanding of the wide confidence intervals for the specificity and NPV when this very low prevalence). Furthermore, in fact the PPV and NPV we are reporting are essentially posterior probabilities (the only problem is that the very low prevalence of resistance makes the confidence interval of the specificity and NPV very wide). Accordingly, in terms of Bayesian posterior probabilities the Prior is the susceptibility in the population (and resistance to these antimicrobials are truly extremely rare, so our dataset accurately represents this so the Prior probability of susceptibility is

already appropriately considered). Based on this, only the posterior probability that an isolate is truly susceptible when the SensiTyper predicted susceptibility can be calculated, which is 100% (basically the PPV). Likewise, the posterior probability that an isolate is truly resistant when the SensiTyper predicted resistance can be calculated and this is also 100% (basically the NPV). Accordingly, mathematically we are already basically presenting the Bayesian posterior probabilities because they are the same as the PPV and NPV in this case, and adding explicit Bayesian posterior probabilities would simply duplicate information and not solve the problems with the wide confidence intervals. Instead for further clarification, we have in the revised manuscript added all the actual numbers in Table 2 (including the very low number of true negatives, i.e., resistant isolates), an additional footnote to Table 2 (explaining the reason for the wide confidence interval for the specificity, NPV and VME rates in the prediction of susceptibility), and mentioned both in the Results section and Discussion section (among the limitations) that nearly all isolates were susceptible to these antimicrobials, which results in wide confidence intervals for the specificity, NPV and VME rates. We think this makes it sufficiently clear, and think readers will fully understand all this, and we really hope this is acceptable for the Reviewer.

Reviewer #4 (Remarks on code availability):
"The github repo is neatly organised and well documented."

Authors' response: We are grateful for the positive response regarding our code availability.

Reviewer #5 (Remarks to the Author):
"I co-reviewed this manuscript with one of the reviewers who provided the listed reports. This is part of the Nature Communications initiative to facilitate training in peer review and to provide appropriate recognition for Early Career Researchers who co-review manuscripts."

Authors' response: We are grateful for the positive response regarding our submitted manuscript and we think this initiative by Nature Communications is great and most valuable.
