# Supplementary material for: Genomic surveillance meets clinical practice: rule-based individualized treatment prediction for gonorrhoea using SensiTyper
Source: Nat Commun. 2026 Oct 30;17:11293. doi: 10.1038/s41467-026-79037-z (PMC13722691; doi:10.1038/s41467-026-79037-z)
Supplement: Supplementary file 4 [file 41467_2026_79037_MOESM2_ESM.docx]

**Supplementary Data. Whole-genome sequencing data for *Neisseria gonorrhoeae* isolates (from 1928-2024) were compiled from 32 published studies, in addition to 568 contemporary isolates (from 2017-2024) sequenced in the present study.** The dataset covers 68 countries and comprises 13,701 gonococcal genomes, representing the largest international collection assembled for genomic antimicrobial susceptibility prediction analyses.
